# Machine Learning‐Enhanced Optimization for High‐Throughput Precision in Cellular Droplet Bioprinting

**DOI:** 10.1002/advs.202412831

**Published:** 2025-04-27

**Authors:** Jaemyung Shin, Ryan Kang, Kinam Hyun, Zhangkang Li, Hitendra Kumar, Kangsoo Kim, Simon S. Park, Keekyoung Kim

**Affiliations:** ^1^ Department of Biomedical Engineering Schulich School of Engineering University of Calgary Calgary Alberta T2N 1N4 Canada; ^2^ Department of Electrical and Software Engineering Schulich School of Engineering University of Calgary Calgary Alberta T2N 1N4 Canada; ^3^ Department of Mechanical and Manufacturing Engineering Schulich School of Engineering University of Calgary Calgary Alberta T2N 1N4 Canada; ^4^ Department of Biosciences and Biomedical Engineering Indian Institute of Technology Indore Indore Madhya Pradesh 453552 India

**Keywords:** bioprinting, cellular droplets, machine learning, optimization

## Abstract

Organoids produce through traditional manual pipetting methods face challenges such as labor‐intensive procedures and batch‐to‐batch variability in quality. To ensure consistent organoid production, 3D bioprinting platforms offer a more efficient alternative. However, optimizing multiple printing parameters to achieve the desired organoid size remains a time‐consuming and costly endeavor. To address these obstacles, machine learning is employed to optimize five critical printing parameters (i.e., bioink viscosity, nozzle size, printing time, printing pressure, and cell concentration), and develop algorithms capable of immediate cellular droplet size prediction. In this study, a high‐throughput cellular droplet bioprinter is designed, capable of printing over 50 cellular droplets simultaneously, producing the large dataset required for effective machine learning training. Among the five algorithms evaluated, the multilayer perceptron model demonstrates the highest prediction accuracy, while the decision tree model offers the fastest computation time. Finally, these top‐performing machine learning models are integrated into a user‐friendly interface to streamline usability. The bioprinting parameter optimization platform develops in this study is expected to create significant synergy when combined with various bioprinting technologies, advancing the scalable production of organoids for a range of applications.

## Graphical abstract

A high‐throughput droplet‐based cellular bioprinting platform enhanced with machine learning optimizes five key printing parameters—bioink viscosity, nozzle size, printing time, printing pressure, and cell concentration—to enable accurate prediction of droplet size. This integrated approach improves precision, reproducibility, and scalability in organoid production while reducing time and material requirements.

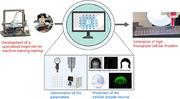

## 1 Introduction

Bioprinting has the potential to replace 2D cell cultures by more closely replicating the 3D microtissue environment.^[^ ^1^, ^2^ ^]^ Moreover, its versatility enables a broad spectrum of applications, spanning tissue engineering and precision medicine,^[^ ^3^, ^4^ ^]^ transplantation,^[^ ^5^ ^]^ pharmaceuticals and high‐throughput screening,^[^ ^6^ ^]^ and cancer research.^[^ ^7^ ^]^ Among many types of bioprinting, pneumatic extrusion bioprinting offers significant advantages in cell‐laden droplet bioprinting, leveraging its inherent simplicity and agility. In recent years, research on stem cell‐derived organoid bioprinting, where the organoids take on a hemispherical shape when placed on a substrate, has seen significant growth.^[^ ^8^, ^9^, ^10^ ^]^ Lawlor et al. bioprinted kidney organoids on a Transwell filter for culture and conducted a comparative analysis with manually created organoids to assess the potential of bioprinting technology,^[^ ^11^ ^]^ while similarly, Shin et al. bioprinted 2 µL cellular droplets containing 8 × 10^3^ nephron progenitor cells to successfully make kidney organoids by utilizing an in‐house modified, low‐cost 3D bioprinter.^[^ ^12^ ^]^ Additionally, when bioprinting with a bioink that combines cells and biomaterials, the hydrogel protects the cells and provides a more native‐like microenvironment, allowing cells to grow and thrive within the 3D structure. Sakthivel et al. utilized inkjet bioprinting to create droplet‐shaped structures with a gelatin methacrylate (GelMA) hydrogel precursor combined with cells, analyzing cell responses to an elastic composite substrate that was periodically stretched.^[^ ^13^ ^]^

The common challenges frequently encountered in these studies include the intricate process of achieving consistent microscale droplets of uniform size. Additionally, optimizing numerous bioprinting parameters, such as temperature, bioink properties, printing time, printing speed, nozzle size, and dispensing pressure, adds to the complexity of the process.^[^ ^14^ ^]^ The quality of bioprinted outcomes is significantly influenced by these parameters, as even minor changes can alter the final result.^[^ ^15^, ^16^ ^]^ Overcoming these obstacles traditionally requires extensive experimentation involving significant human resources and large quantities of bioink materials for trial and error. This challenge is further amplified when working with stem cells, which are notably more sensitive than other cell sources such as immortalized cell lines and primary cells.^[^ ^17^ ^]^ The use of stem cells highlights the critical importance of achieving precise control over droplet size, as even minor variations can significantly impact cell viability, functionality, and degree of maturity.^[^ ^18^ ^]^ Therefore, several studies have been conducted to optimize and generalize printing parameters. Webb et al. developed a generalized evaluation method, termed Parameter Optimization Index, for assessing bioprinted samples. This method utilizes various bioprinting parameters, including biomaterial composition, nozzle size, printing speed, and printing pressure.^[^ ^19^ ^]^ Specifically, many studies were found where machine learning (ML) was applied to bioink optimization. Bone et al. utilized ML models to improve the printing fidelity of alginate hydrogels by generating a dataset through adjustments to parameters such as inkjet concentration, nozzle diameter, and printing speed. Ultimately, the hierarchical ML model demonstrated better predictive performance than conventional neural networks, effectively explaining the relationship between output variables and print quality.^[^ ^20^ ^]^ Lee et al. introduced multiple regression ML models to design bioinks with distinct characteristics of atelocollagen and native collagen, ultimately developing inks that satisfy both shape fidelity and biocompatibility.^[^ ^21^ ^]^ In addition, James et al. investigated the relationships between changes in mechanical properties, degradation, and swelling ratio by optimizing the hydrogel composition parameters.^[^ ^22^ ^]^ They found that printing parameters were primarily influenced by the composition of the hydrogels used. However, the studies mentioned above primarily focused on optimizing printing parameters, leaving greater potential for approaches that integrate a broader range of technologies to efficiently enhance the organoid bioprinting process.

In this study, we developed a novel bioprinter designed to address the challenges of high‐throughput and precise 3D cellular droplet bioprinting. The system is equipped with a large‐scale data collection capability tailored for traditional ML and deep learning (DL) applications, enabling efficient optimization of bioprinting parameters. With precise control over micro‐scale droplet volumes and compatibility with various bioinks, the bioprinter supports the production of cellular droplets at high‐throughput. This versatile technology has the potential to be applied to a wide range of existing bioprinting systems.

**Figure 1:**
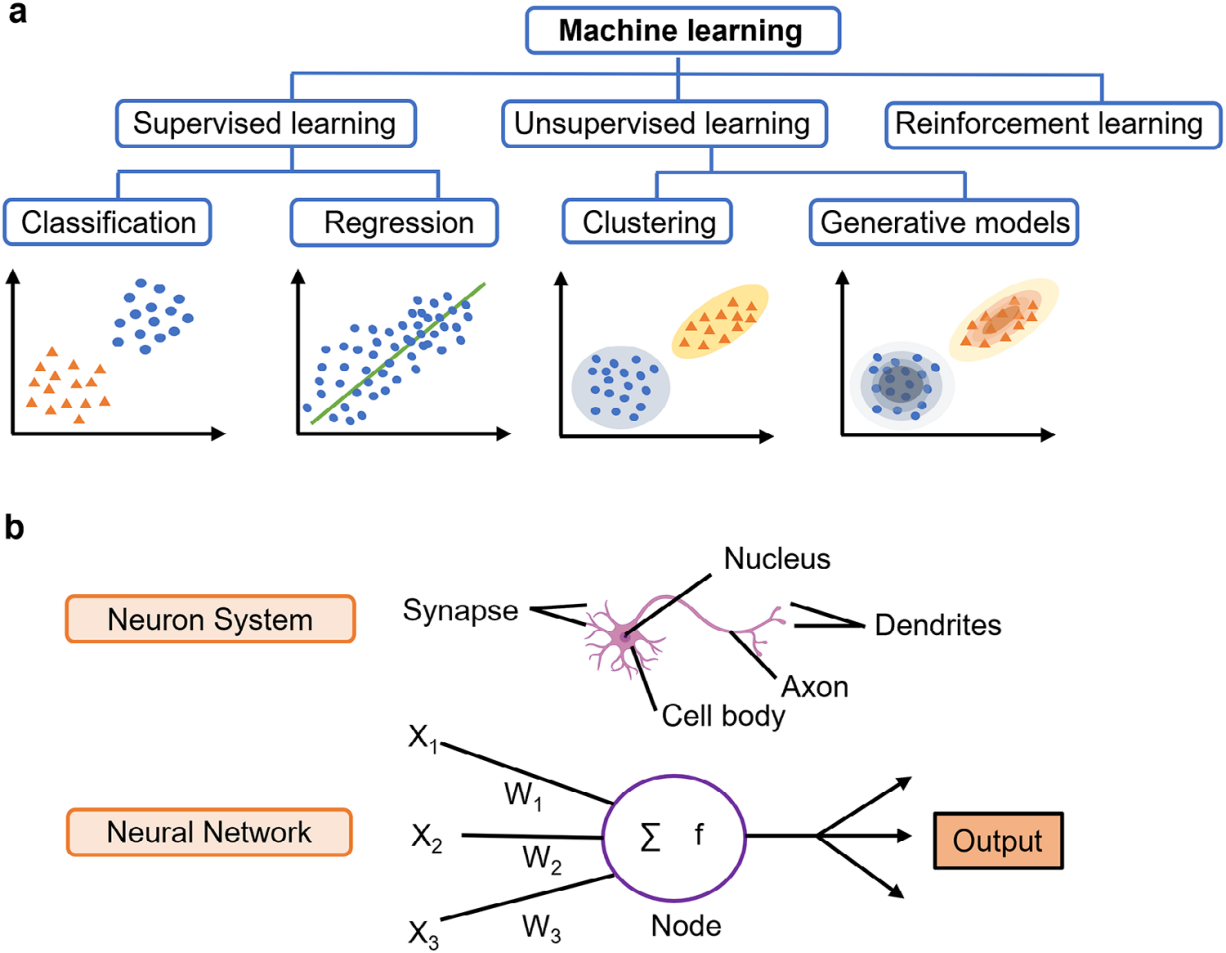
Overview of machine learning types and neural network structures. a) Machine learning encompasses various approaches, including supervised learning (e.g., regression and classification), unsupervised learning (e.g., clustering and generative models), and reinforcement learning. b) Neural network structures are inspired by the architecture and functionality of the human nervous system.

The combination of ML with advanced bioprinting technology can potentially accelerate research in tissue engineering and precision medicine.^[^ ^23^ ^]^ ML refers to computer programs that based on big data, autonomously learn to predict the future or make decisions.^[^ ^24^ ^]^ It is an artificial intelligence (AI) paradigm that goes beyond simple data training, continuously collecting and learning to enhance accuracy. Wu and Xu utilized ensemble learning to predict the velocity and volume of droplets generated during the inkjet‐based bioprinting process.^[^ ^25^ ^]^ However, the authors noted that additional experiments are required to collect a broader range of parameters for further improvement. In the development principles of ML, there are three main paradigms: supervised learning, which involves training on labeled datasets; unsupervised learning, which processes unlabeled data to discover hidden patterns or structures within the dataset; and reinforcement learning, where algorithms learn through feedback in the form of rewards or penalties (**Figure** **1**a).^[^ ^26^ ^]^ DL is a subset of ML that is based on artificial neural networks, which are similar to the human nervous system (Figure 1b).^[^ ^27^ ^]^ Therefore, in this study, the section of ML that excludes DL will be referred to as traditional ML. One key difference between ML and DL is that DL can collect and process data without undergoing the data preprocessing tasks typically required in traditional ML (Table S1, Supporting Information).^[^ ^28^, ^29^ ^]^ Verheyen et al. used supervised ML, which can identify patterns within given data, to construct predictive frameworks with material databases and assessed the predictability of soft granular material design spaces.^[^ ^30^, ^31^ ^]^

To provide users with enhanced controllability and printability, we established a high‐throughput bioprinting platform incorporating data‐driven traditional ML and DL for bioprinting the array of cellular droplets including organoids. We developed a customized 3D bioprinter with a high throughput droplet image acquisition system for the thousands of printed droplets based on various bioprinting parameters (e.g., bioink viscosity, nozzle size, printing time, printing pressure, and cell concentration). We also developed software to automatically measure droplet volume using image processing and transferred the data into the ML algorithms. Ultimately, we created a web‐based user interface that leverages well‐trained ML algorithms to enable users with limited experience in bioprinting to easily perform organoid printing. The combination of ML with advanced bioprinting technology can potentially accelerate research in creating various organoids.^[^ ^23^ ^]^ In addition, the approach utilizing traditional ML and DL to optimize bioprinting parameters will significantly reduce human resources and costs in the bioprinting process.^[^ ^32^ ^]^ Finally, our ML‐integrated bioprinting system promises to simplify printing procedures, leading to swifter and more effective outcomes.

## 2 Results

### 2.1 Mechanical and Physiochemical Properties Investigation of GelMA‐Alginate Bioinks

Achieving a successful bioprinted scaffold depends on biomaterials with optimized rheological properties. Rheological assessments were conducted on various biomaterial combinations to evaluate their suitability for long‐term cell encapsulation within hydrogels. These hydrogel precursors were tested for rheological characterization, including crosslinking kinetics and static viscosity measurements.

**Figure 2:**
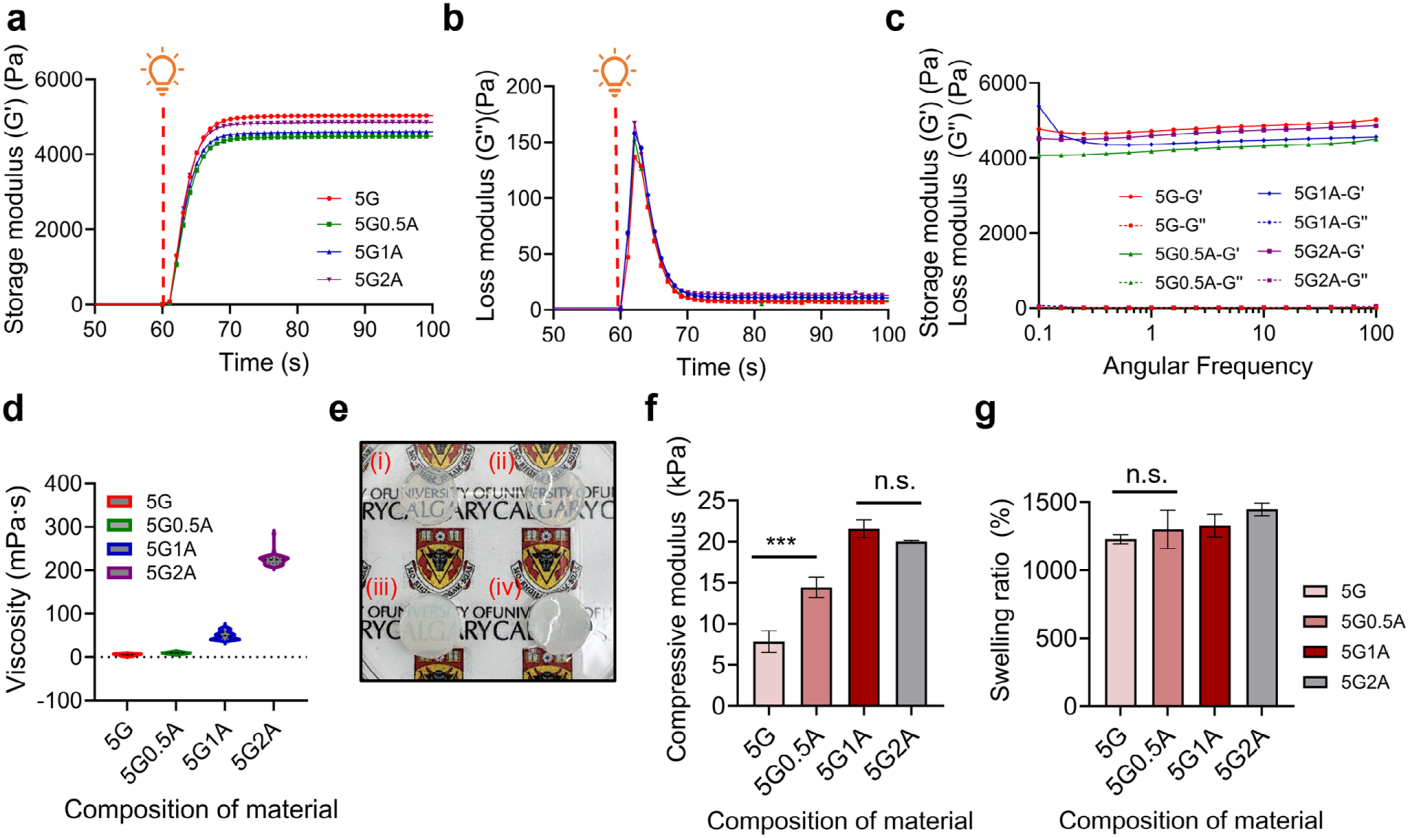
Rheological, mechanical, and physical characterization of GelMA‐alginate hydrogels. a) Storage modulus measured at the initiation of light exposure after 60 sec. b) Loss modulus measured at the initiation of light exposure after 60 sec. c) Angular frequency response assessing the crosslinking stability of materials. d) Static viscosity of materials used as inputs for bioprinting parameters. e) Optical transmittance of specimens subjected to photocrosslinking under 405 nm wavelength light. f) Compressive modulus for four distinct material formulations. (n.s. not significant, ^***^ *p* < 0.001). g) Swelling ratio for four distinct material formulations.

**Figure** **2**a illustrates the rheological behavior of various GelMA‐alginate compositions during the photocrosslinking process. The bioinks used in this study are designated as 5G, 5G0.5A, 5G1A, and 5G2A, where 5G denotes a formulation with 5% (w/v) GelMA, and 0.5, 1, and 2A indicate the respective concentrations (w/v) of alginate (0.5, 1, and 2%) added to 5% GelMA. These notations are used consistently throughout the manuscript to describe the GelMA‐alginate formulations.

The 5% (w/v) GelMA (5G) formulation exhibited the highest storage modulus upon light exposure, indicating superior elastic properties and robust network formation. This suggests that the 5G precursor achieves the most efficient crosslinking, resulting in a mechanically stronger hydrogel structure. Samples with a high concentration of methacryloyl groups demonstrated a rapid initiation of the photocrosslinking reaction, as evidenced by the immediate increase in storage modulus. This behavior is characteristic of highly functionalized GelMA, where the abundance of crosslinkable groups promotes rapid network formation. As the alginate concentration increased, the onset of crosslinking was delayed. However, the storage modulus exhibited higher values, corresponding to increased viscosity. This phenomenon is likely due to the physical interference of alginate molecules with the GelMA photocrosslinking process. Alginate molecules may impede interactions between methacryloyl groups, resulting in a less densely crosslinked network and, consequently, lower elastic properties.

As the alginate concentration increased, the loss modulus appeared to slightly increase (Figure 2b). This trend aligns with the decrease in storage modulus, as the alginate is thought to interfere with the photocuring process. The 5G2A formulation exhibits a higher loss modulus, indicating greater viscosity and reduced elasticity compared to 5G. The rate of decrease in loss modulus during photocuring appears slower for samples with higher alginate concentrations. This reflects the interference of alginate with the crosslinking process, resulting in a more gradual transition from viscous to elastic behavior. The initial loss modulus (before initiating the light exposure) tends to be higher for samples with higher alginate concentrations due to the increased viscosity contributed by the alginate polymer. These findings highlight the complex interplay between GelMA concentration, methacryloyl group density, and the presence of secondary polymers like alginate in determining the final mechanical properties of photocrosslinked hydrogels. Such insights are critical for optimizing bioink formulations to achieve specific mechanical characteristics tailored to various bioprinting applications.

As shown in Figure 2c, the storage modulus remains relatively constant across a range of angular frequencies, indicating that the material has formed a stable and elastic network. All materials used in the test here are fully crosslinked and exhibit a plateau in storage modulus values, demonstrating that the deformation rate does not significantly influence the material's elastic properties. In addition, for a fully crosslinked material, the loss modulus is significantly lower than the storage modulus across the same range of angular frequencies. This suggests that the material behaves predominantly as a solid (elastic) than a liquid (viscous). The degree of crosslinking in GelMA hydrogels can be effectively assessed by analyzing angular frequency trends, the immediate response to light exposure, and the relative stability of the storage modulus and loss modulus.

Additionally, the static viscosity of the material, a critical bioprinting parameter, was investigated. During this measurement, a fluid typically exhibits a viscosity plateau at lower shear rates and then displays shear thinning as the shear rate increases, forming a flow curve. Alginate, a high molecular weight polysaccharide, increases the average molecular weight of the entire hydrogel precursor when added to a GelMA solution. This results in greater entanglement between polymer chains, which significantly contributes to the increased viscosity of the solution. As illustrated in Figure 2d, the static viscosity of the GelMA based hydrogel precursor increases with higher alginate concentrations. Such high viscosity positively impacts printing fidelity and the ability to maintain the structural integrity of the printed constructs.^[^ ^33^, ^34^ ^]^

To evaluate the transparency, the logo remained visible in the 5G and 5G0.5A samples (Figure 2e). Conversely, the 5G1A sample showed reduced visibility of the logo, while the 5G2A sample exhibited complete opacity, fully obscuring the underlying logo. These observations indicate a progressive increase in opacity correlated with compositional variations among the samples, with a notable rise in opacity as the alginate ratio increases.

As the alginate concentration increases, the initial mechanical strength of the hydrogel is enhanced. However, excessive alginate concentrations may interfere with the photocrosslinking of GelMA. Figure 2f shows that the mechanical strength increases up to the 5G1A composition but slightly decreases for 5G2A. This suggests that alginate could obstruct light transmission during the photocrosslinking process or interfere with the interaction of GelMA's methacrylate groups.

Due to alginate's high hydrophilicity, its incorporation into GelMA enhances the hydrogel network's porosity, generally leading to an increased swelling ratio.^[^ ^35^, ^36^ ^]^ As shown in Figure 2g, the swelling ratio increases with the addition of alginate to the 5G formulation.

### 2.2 Development and Validation of the Developed Bioprinting Platform

A conversion of a commercially available 3D printer into a bioprinter is detailed in Shin et al.^[^ ^12^ ^]^ This modified system was based on a low‐cost 3‐axis fused deposition modeling printer (Ender 3 Pro, Creality 3D, China) integrated with a pneumatic‐based liquid dispenser (FEITA 983, FEITA, China). We designed and implemented a printed circuit board (PCB) using a converter to change the 12 V output from a standard 3D printer fan to a 5 V output, with a MOSFET to switch on and off. The PCB features a fast‐switching circuit (Figure S1a, Supporting Information), a programmable linear stage (Figure S1b), LED lighting and a cell mixer (Figure S1c, Supporting Information), and a modified liquid dispenser (Figure S1d, Supporting Information).

**Figure 3:**
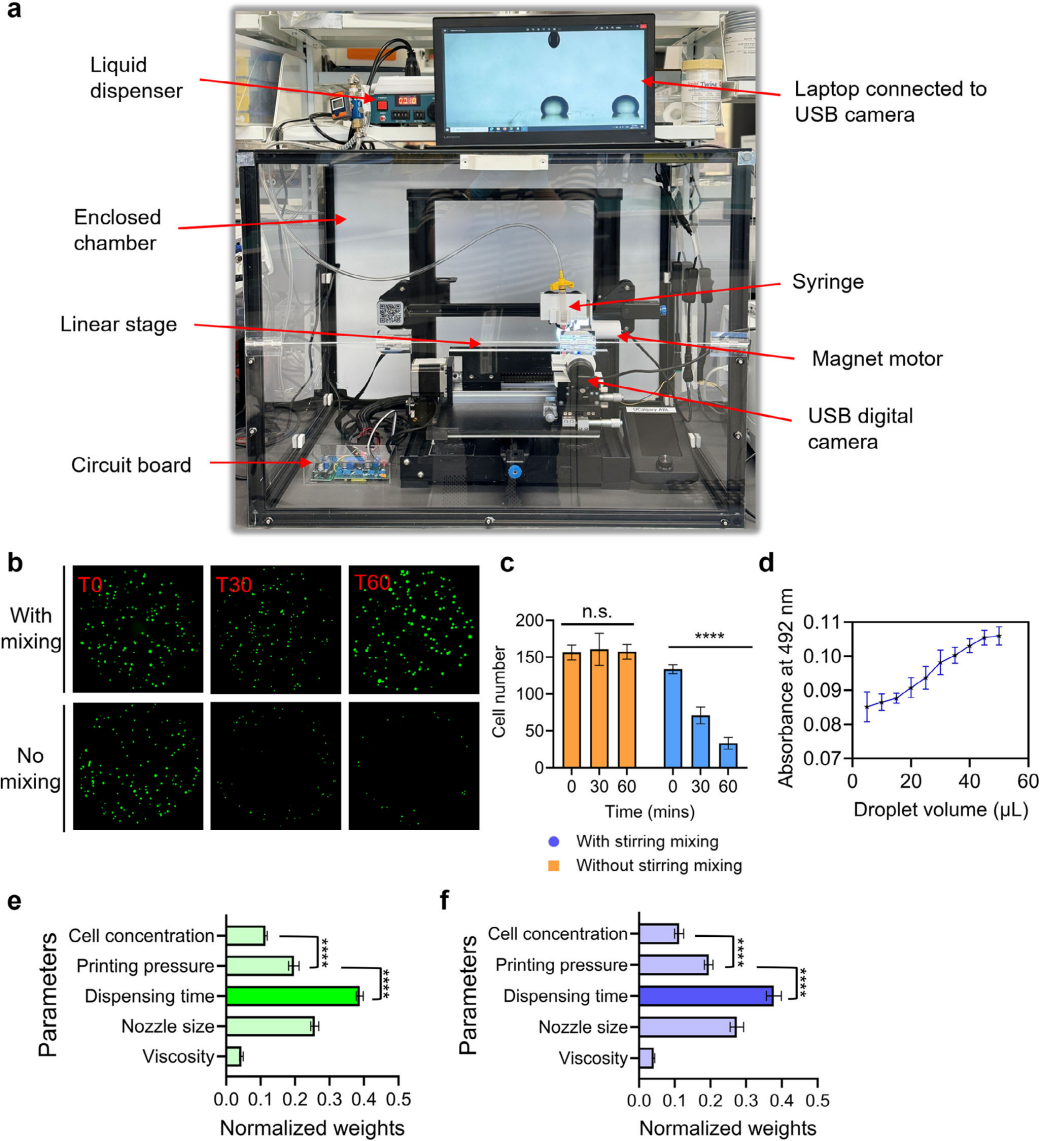
a) Modified and developed 3D bioprinting setup. b) Impact of in‐syringe bioink homogenization on cell distribution and viability in bioprinted constructs. c) Comparison of bioprinted cell numbers over time intervals (0, 30, 60 min) to assess consistency in cell numbers with and without the stirring magnet (n.s.: not significant, ^****^ *p* < 0.0001). d) Absorbance results at 492 nm correlated with cell numbers resulting from droplet volume variations. e) Feature weights derived from the decision tree algorithm (^****^ *p* < 0.0001). f) Feature weights derived from the random forest algorithm (^****^ *p* < 0.0001).

To accommodate the data‐intensive nature of ML training, a movable printing stage along with additional modifications was incorporated into the original 3D bioprinting platform (**Figure** **3**a). The G‐code was programmed to move the bed stepper motor, shifting the printing linear stage to the right after each droplet deposition, thereby facilitating the acquisition of new cell‐laden droplet image data (Movie S1, Supporting Information). A USB digital camera (Jiusion, Shenzhen, China) was integrated and connected to a laptop to capture images of the droplets, enabling real‐time monitoring and analysis (Movie S2; Figure S2, Supporting Information). This integration provides a detailed visualization of the droplets. The syringe was connected to a liquid dispenser, and the setup employed a modified circuit board to ensure precise control over the air pressure output. Using the pneumatic extrusion‐based bioprinter we developed, it was possible to perform bioprinting with significantly smaller droplets of 0.1 µL compared to piston, screw, or syringe pump‐based methods (Movie S3, Supporting Information). Overall, this system demonstrated precise control over various droplet sizes with high repeatability. This configuration ensures that the developed bioprinting system consistently maintains the predetermined air pressure settings throughout the entire process. For consistent cell number bioprinting, we developed a motorized button capable of inserting a 10 × 1.5 mm stir bar into the syringe to mix the bioink homogenously (Movie S4, Supporting Information). The video demonstrates the process of loading a mixture of phosphate‐buffered saline (PBS) and pink glitter into a syringe for visualization purposes, followed by stirring.

In Figure 3b, the dispensed bioink was immediately crosslinked and imaged under a microscope at three time points: at the start of printing (T0), 30 min later (T30), and 60 min later (T60) (Movie S5, Supporting Information). These results illustrate that the mixing system maintained a constant cell number over time, whereas droplets dispensed without the mixing system exhibited a decrease in cell number over time (Figure 3c). To comprehensively analyze the effects of two independent variables (the presence of a magnetic stirring system and printing duration) on a single dependent variable (cell number), we employed two‐way analyses of variance (ANOVA). This statistical method facilitates the simultaneous examination of the main effects of each independent variable as well as their potential interaction effect on the cell number.

The printed green fluorescent protein (GFP)‐tagged 3T3 cell‐laden droplets were produced in various sizes (up to 50 µL) on a 96‐well plate. The fluorescence signal was measured at an absorbance of 492 nm using a plate reader to quantify the intensity of fluorescence emitted by samples containing the GFP‐tagged cells. By measuring the fluorescence intensity, the plate reader provided quantitative data on the number of cells present in each droplet. The results demonstrated that as the droplet size increased, the fluorescence signal also increased, corresponding to a higher number of cells (Figure 3d). This indicates that the developed bioprinting system not only produces uniform and stable droplets but also automates the data collection process, enabling high‐throughput analysis of multiple samples simultaneously. This efficiency is particularly beneficial in experiments requiring large datasets for ML training.

Additionally, this study focused on identifying and quantifying the relative importance of bioprinting parameters in determining the final volume of bioprinted droplets (**Table** **1**). Using a systematic approach, each bioprinting parameter was varied independently while others were held constant to assess its effect on droplet volume. All tested parameters have a statistically significant effect on droplet volume (Table S2, Supporting Information). The weights shown in Figure 3e,f were derived from feature importance metrics calculated using the Random Forest Regressor class in the scikit‐learn library as explained in the method section. These weights represent the relative contribution of each parameter to improving the prediction accuracy of droplet volume outcomes. This analysis highlights the factors most critical to achieving high fidelity in bioprinting. Among the parameters, dispensing time emerged as the most influential factor in determining droplet volume, followed by nozzle size and printing pressure. Under the assumption that the primary parameters are precisely controlled, the study revealed that material viscosity and cell concentration exert relatively minor effects on droplet volume regulation. These findings provide valuable insights into the hierarchical importance of bioprinting parameters, enabling more precise control over droplet volume in bioprinting processes. Such knowledge is crucial for optimizing bioprinting protocols and achieving higher levels of precision in fabricating complex tissue constructs.

**Table 1:** Bioprinting parameters used to achieve the desired droplet volume include biomaterial viscosity, nozzle size, printing time, printing pressure, and cell concentration.

Independent variables					Dependent variables
Viscosity (mPa·s)	Nozzle size (I.D.^a)^ mm)	Printing time (s)	Printing pressure (psi)	Cell concentration	Droplet volume (µL)
10.03 24.66	23 (0.337) 25 (0.26) 27 (0.21)	0.05 0.1 0.15	1.5 2	2.8 × 10^6^/3 mL 5.6 × 10^6^/3 mL	–

^a)^ I.D.: inner diameter

### 2.3 Image Processing and Droplet Volume Calculation

**Figure 4:**
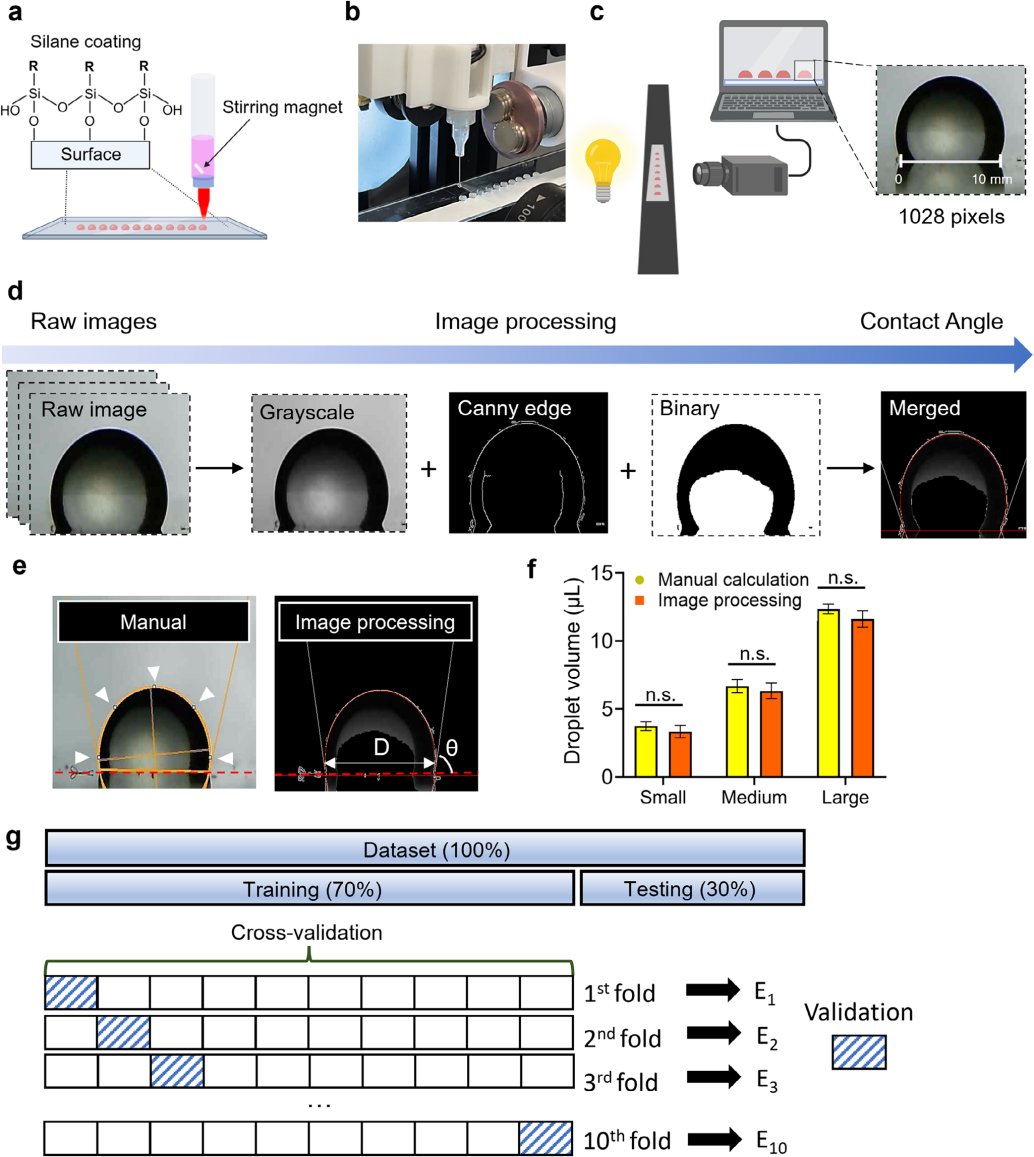
a) High‐throughput bioprinting multiple cellular droplets during a single bioprinting run on a silane‐coated glass slide. b) Demonstration of consistency in bioprinted GFP‐tagged 3T3 fibroblast cellular droplets. c) Illustration of the calibration method for measuring droplet volume in the droplet volume measurement system. d) Overview of the image preprocessing pipeline for preparing the dataset for droplet volume calculation. e) Comparison of droplet volumes manually calculated using ImageJ (left) and measured using the developed image processing method (right), (D: diameter). f) Comparative analysis of manual and image processing‐based droplet size measurements across three size categories. (*n* = 10) (n.s.: not significant). g) Data partitioning and validation strategy: An initial 70:30 dataset split into training and testing sets, followed by 10‐fold cross‐validation on the training set to mitigate overfitting.

To achieve uniform droplet volume during bioprinting and maintain consistent printing conditions, the substrate of the glass slide was treated with a hydrophobic silane coating (**Figure** **4**a). This treatment enabled the generation of high‐throughput, uniform droplets on a standard microscopy glass slide. Specifically, 1H,1H,2H,2H‐Perfluorooctyltrichlorosilane (Fisher Scientific, Waltham, MA, USA) was used to coat the surface of the glass slides, producing hydrophobic surfaces. The glass slides were then placed on a movable linear stage, facilitating droplet formation. Cell‐laden bioinks (5G1A and 5G0.5A) were loaded in a 3 mL syringe equipped with a small magnetic stir bar to ensure homogeneous cell distribution in the bioink throughout the droplet printing process (Figure 4b).

A micro ruler was placed at the droplet dispensing location to precisely measure the actual length of the droplet at the dispensing point, yielding a value of 10 mm. The corresponding pixel count for this length in the images was found to be 1028 pixels (Figure 4c **)**. This calibration resulted in a conversion factor of 0.009728 mm per pixel, enabling the accurate translation of pixel measurements into actual dimensions.

To outline the development of our custom droplet size calculation software, the image processing pipeline incorporates a series of techniques to enhance efficiency and accuracy. Precise edge detection was essential to improve droplet volume calculation accuracy. Raw images underwent three extraction techniques (e.g., grayscale conversion, Canny edge detection, and binary thresholding), which were combined to produce the final image (Figure 4d). Initially, grayscale conversion was applied to reduce computational complexity by transforming color images into single‐channel representations. Next, Canny edge detection was used to identify image boundaries and obtain clear outlines. Finally, binary thresholding simplified the image to black and white pixels, effectively reducing noise and irrelevant details. By employing these techniques sequentially, the image processing pipeline achieved both simplification of color images and enhanced processing efficiency, providing a robust foundation for subsequent analysis tasks.

In Figure 4e, the manual droplet volume measurement was performed by analyzing the raw image in Figure 4d using ImageJ, where five points were manually placed along the droplet boundary and the volume was calculated individually for each droplet. In contrast, the automated droplet size calculation method developed in this study followed a systematic procedure in Python. After undergoing image processing in Figure 4d, the merged image was used for calculation analysis, followed by circle detection using the HoughCircles function from Python's OpenCV library. The detected circles were then utilized to define the droplet boundary based on the equation of a circle centered at (*h,k*) with radius *r* (Figure S3, Supporting Information). A horizontal line *y = a* (slope = 0) was then applied to calculate the intersection points *A* and *B*. The distance between these two points was measured to determine the droplet diameter, and the contact angle was obtained by calculating the angle between the tangent line at the intersection points and the horizontal reference line *y = a*. Finally, the diameter and contact angle were used in Equation (1) to automatically calculate the droplet volume, enabling high‐throughput analysis of multiple droplet images simultaneously. This calculation was performed using Equation (1):

(1) $$V=\frac{\pi \;D^{3}}{24}\left(\frac{2-3\cos\theta +\cos^{3}\theta }{\sin^{3}\theta }\right)$$

where *r* is the radius of the droplet base (in mm), *h* is the height of the droplet (in mm), *V* is the calculated droplet volume (in µL), and *θ* is the contact angle between the droplet and the substrate (in degrees).^[^ ^37^ ^]^

Both measurements for this analysis were obtained from 10 randomly selected droplets from each size group (small, medium, and large), resulting in a total of 30 images. These images were chosen to validate the droplet volume measurement method and provide a representative subset of the 1758 data points. The selection was based on clarity and diversity to ensure that the subset effectively captured the range of droplet sizes observed in the full dataset. The analysis encompassed droplets of various sizes: small droplets with a mean volume of 3.54 µL, medium droplets averaging 6.54 µL, and large droplets with a mean volume of 11.98 µL. As a result of the comparative analysis, no statistically significant difference was observed between the droplet volume measurements obtained using the ImageJ software and our custom‐developed droplet size calculation software (Figure 4f).

By combining these processed images, we achieved reliable and consistent volume measurements across all samples, demonstrating the robustness of the proposed approach in capturing the fine details necessary for precise droplet quantification. The droplet volumes obtained were extracted and transferred to excel, where the data was partitioned into training and testing sets to train three traditional ML and two DL algorithms (Figure S4, Supporting Information). The total dataset of 1758 images was divided into a 70% training set and a 30% testing set (Figure 4g). To mitigate overfitting, 10‐fold cross‐validation was performed to ensure the performance estimates were robust and that the models generalized properly to new droplet images. The dataset was randomly divided into 10 equal‐sized subsets or folds, with the model trained on 9‐folds and validated on the remaining 1‐fold. This process was repeated 10 times, with each fold used as the test set once. Finally, the model's performance was averaged across all 10 iterations.

### 2.4 Optimization of the Hyperparameters

Optimal hyperparameter tuning is essential for maximizing the performance of traditional ML models. By leveraging various optimization methods, such as grid search, random search, Bayesian optimization, gradient‐based methods, evolutionary algorithms, and Hyperband, practitioners can efficiently explore hyperparameter options and identify the best configurations for their specific use cases.

To evaluate the performance of the algorithms in predicting printing parameters, our study was structured into two distinct phases: Phase 1, focuses on hyperparameter optimization, and Phase 2, compares the optimized algorithms. In Phase 1, key parameters for each algorithm, such as learning rate, batch size, and tree depth, were systematically tuned to achieve optimal performance. Phase 2 involved assessing the optimized models using metrics like mean absolute error (MAE), root mean square error (RMSE), R‐squared, and computation time to provide a comprehensive comparison of their accuracy and efficiency in predicting printing parameters. This section primarily focuses on Phase 1, which addresses hyperparameter optimization.

**Figure 5:**
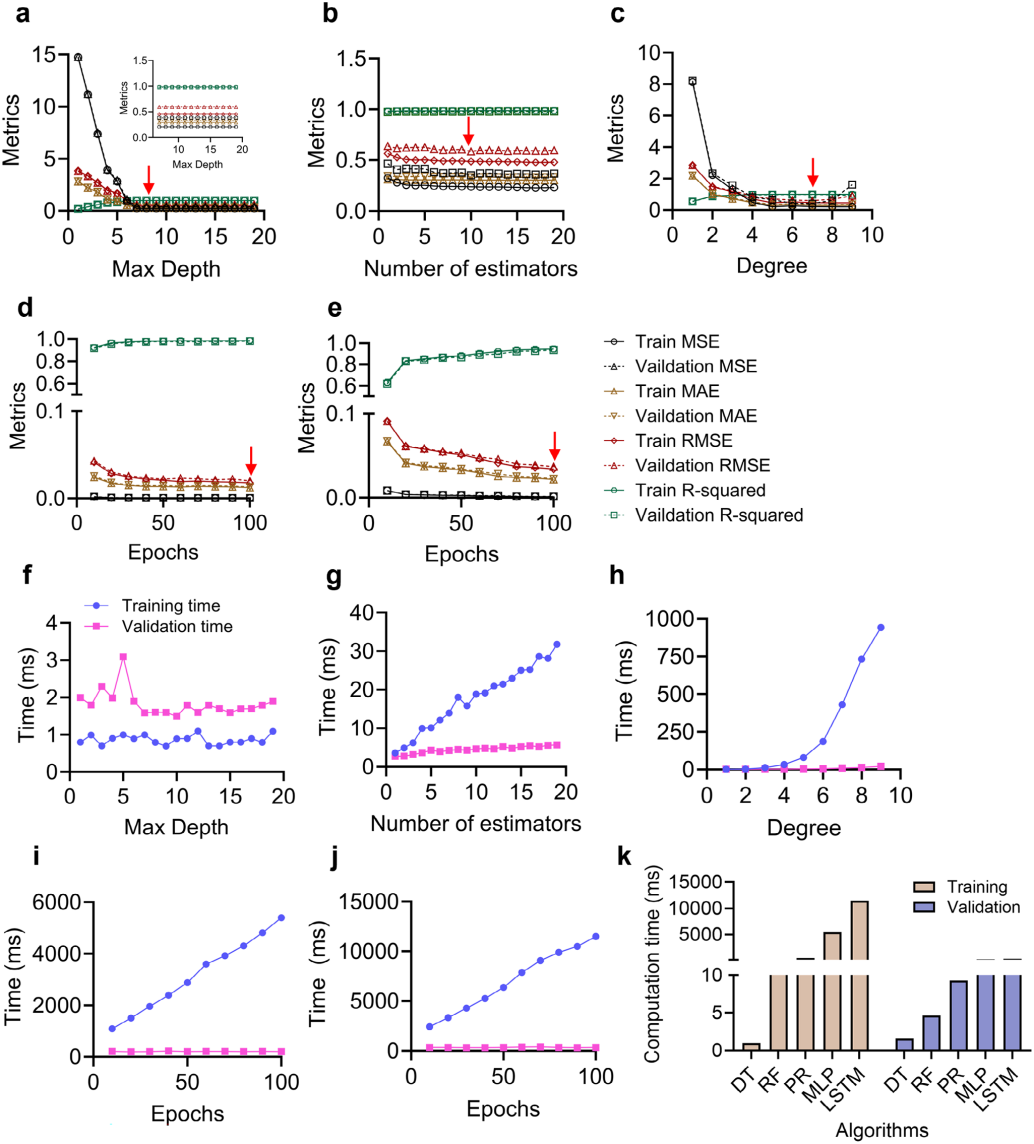
a) Optimization process for identifying the optimized hyperparameters for a decision tree. b) Optimization process for identifying the optimized hyperparameters for a random forest. c) Optimization process for identifying the optimized hyperparameters for a polynomial regression. d) Optimization process for identifying the optimized hyperparameters for a multilayer perception. e) Optimization process for identifying the optimized hyperparameters for a long short‐term memory. f) Training and validation time during hyperparameter optimization of the decision tree. g) Training and validation time during hyperparameter optimization of the random forest. h) Training and validation time during hyperparameter optimization of the polynomial regression. i) Training and validation time during hyperparameter optimization of the multilayer perception. j) Training and validation time during hyperparameter optimization of the long short‐term memory. k) Comparison of computation times for all algorithms under optimized hyperparameter settings.

An optimization process was conducted to determine the optimal hyperparameters for each algorithm. This process involved 10‐fold cross‐validation to identify the hyperparameters that yielded the best performance. K‐fold cross‐validation mitigates bias and variance associated with a single data split, resulting in a more robust evaluation of model performance.^[^ ^38^ ^]^ Additionally, it aids in detecting overfitting issues and facilitates the discovery of a generalized model, with each algorithm exhibiting distinct optimized hyperparameters. In **Figure** **5**a, the decision tree (DT) performed optimally with a maximum depth of 7, while the random forest (RF) demonstrated superior performance with 10 estimators (Figure 5b). Given the multitude of hyperparameters in RF, a systematic approach employing GridSearchCV, an automated hyperparameter optimization tool, was utilized to determine the most optimal combination. Among the identified combinations, the parameter with the highest weighting, namely the number of estimators, was selected as the representative hyperparameter. Deeper architectures can enhance classification accuracy, however, they increase the risk of overfitting, necessitating appropriate regularization. Polynomial regression (PR) yielded optimal results with a degree of 7 (Figure 5c), multilayer perception (MLP) performed best with a maximum epoch of 100 (Figure 5d), and long short‐term memory (LSTM) achieved the peak performance with a maximum epoch of 100 (Figure 5e).

### 2.5 Computation Time for Hyperparameter Optimization

Hyperparameter optimization often involves exploring numerous combinations of hyperparameters. The time required to evaluate each combination directly impacts the overall optimization process. Typically, training times exceed validation times due to the iterative nature of the training process and the complexity of the models involved (Figure 5f–j). Interestingly, the DT model exhibited longer validation times than training times. This anomaly arises because the validation phase, particularly with k‐fold cross‐validation, does not benefit from the same level of parallel processing as the training phase. Consequently, the validation phase can take longer. Despite this, DT algorithms train quickly due to their ability to build multiple trees simultaneously. The DT model achieved hyperparameter optimization with the shortest computation time compared to other algorithms during the optimization process (Figure 5k **;** **Table** **2**).

**Table 2:** Computation times of three traditional machine learning algorithms and two deep learning algorithms using the dataset with optimized hyperparameter. Training and validation times are presented for the 10‐cross‐validation process.

Algorithms	Training time (ms)	Validation time (ms)
Decision tree	0.998282	1.591182
Random forest	18.855333	4.672742
Polynomial regression	430.593181	9.286737
Multilayer perception	5394.694638	205.264473
Long short‐term memory	11 486.32846	335.380983

The complexity of the model, such as the depth of a neural network or the number of trees in an RF, directly impacts the duration of the training. More complex models require additional time to converge to an optimal solution. As a result, deeper models such as MLP and LSTM take longer to train compared to traditional ML algorithms (e.g., DT, RF, and PR) (Figure 5k). In summary, while both training and validation are essential components of hyperparameter optimization, training generally demands more time due to its iterative nature and the need for parameter updates. Conversely, validation is faster as it evaluates the model's performance without further adjustments. Implementing efficient optimization techniques and effective resource management can help balance the time requirements of these phases, ultimately enabling more effective and timely model development.

### 2.6 Comparative Evaluation of Machine Learning Algorithms for Droplet Volume Prediction

**Figure 6:**
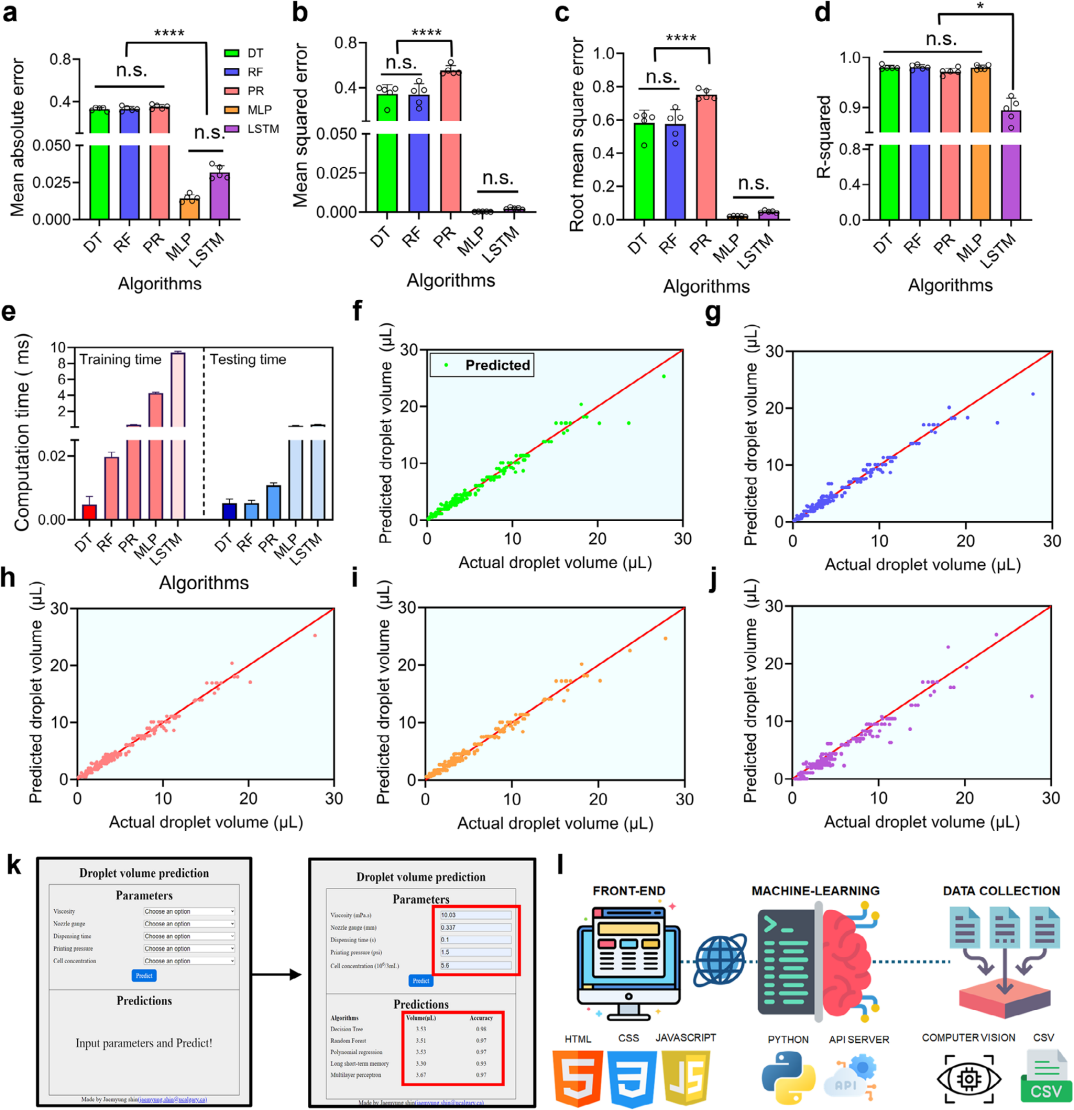
Performance evaluation of traditional machine learning and deep learning models. a) Mean absolute error across models (n.s.: not significant, ^****^ *p* < 0.0001). b) Mean squared error comparison (n.s.: not significant, ^****^ *p* < 0.0001). c) Root mean square error analysis (n.s.: not significant, ^****^ *p* < 0.0001). d) R‐squared values (n.s.: not significant, ^*^ *p* < 0.05). e) Computational time for training and testing phases (*n* = 5). f) Visualization of model performance by comparing the actual and predicted values of the decision tree. g) Random forest: comparison of actual and predicted values. h) Polynomial regression: comparison of actual and predicted values. i) Multilayer perception: comparison of actual and predicted values. j) Long short‐term memory: comparison of actual and predicted values. k) Implementation of a web‐based interface for the droplet volume measurement system, optimized using traditional machine learning and deep learning algorithms. l) Schematic representation of the full‐stack web application architecture.

The hyperparameters identified as optimal during the initial optimization phase were applied to each algorithm. Each model was then subjected to a rigorous training and evaluation process involving 10 independent iterations. To compare the performance of the algorithms, we evaluated three traditional ML and two DL algorithms using the following metrics: MAE, RMSE, and R‐squared (**Table** **3**). MAE serves as a useful indicator for analyzing the model's prediction accuracy and the magnitude of error. As shown in **Figure** **6**a, there was no statistically significant difference among the traditional ML algorithms (DT, RF, and PR) or between the DL algorithms (MLP and LSTM). However, a statistically significant difference was observed between the traditional ML and DL algorithms, with a *p*‐value of 0.0000108. This result indicates that the MLP and LSTM models achieved smaller MAE values than the DT, RF, and PR models, meaning their predictions were closer to the actual values. Consequently, MLP and LSTM demonstrate superior prediction accuracy and overall performance.

**Table 3:** Performance evaluation of three traditional machine learning algorithms and two deep learning algorithms.

Algorithms	Mean Absolute Error (MAE)	Root Mean Square Error (RMSE)	R‐Squared
Decision tree	0.329 ± 0.017	0.582 ± 0.077	0.980 ± 0.004
Random forest	0.332 ± 0.022	0.576 ± 0.087	0.981 ± 0.005
Polynomial regression	0.511 ± 0.211	0.750 ± 0.036	0.968 ± 0.005
Multilayer perception	0.014 ± 0.002	0.021 ± 0.002	0.980 ± 0.005
Long short‐term memory	0.032 ± 0.004	0.048 ± 0.007	0.895 ± 0.024

Second, mean squared error (MSE) is calculated by averaging the squared differences between predicted and actual values. Lower MSE values indicate a closer alignment between model predictions and actual values. The DL models, MLP and LSTM, demonstrated the lowest MSE values with no statistically significant difference, indicating their superior performance (Figure 6b). MSE is particularly sensitive to outliers because it uses squared differences in its calculations. The PR model, which exhibited relatively higher MSE values, may have captured outliers less effectively than the other algorithms. However, it is noteworthy that all models in this study achieved MSE values below 1, suggesting generally good performance across the board.^[^ ^39^ ^]^ It is important to consider that, while MSE provides valuable insights into model performance, it should be interpreted in conjunction with other metrics to comprehensively evaluate model efficacy.

Third, RMSE mitigates the distortion caused by squaring the errors in MSE by taking the square root, making it more interpretable. Unlike MSE, RMSE provides a more intuitive measure of error in the same units as the predicted variable, making it easier to interpret in practical applications. As shown in Figure 6c, the DL models (MLP and LSTM) exhibit the lowest RMSE values, indicating superior performance. For traditional ML models, no statistically significant difference was observed between the DT and RF models. However, a statistically significant difference was observed between the PR model. Notably, all models evaluated in this study achieved RMSE values below 1. Together, MSE and RMSE complement each other by offering insights into both the overall distribution of errors and their practical implications. MSE is particularly suited for evaluating models where minimizing large deviations is crucial, while RMSE provides a more intuitive assessment of general error magnitudes in real‐world scenarios.

Finally, R‐squared is an effective evaluation metric for comparing relative performance, as it represents the proportion of variance in the actual values explained by the predicted values. The closer the R‐squared value is to 1, the better the model's performance; conversely, a value closer to 0 indicates a less accurate model.^[^ ^39^ ^]^ As shown in Figure 6d, the models DT, RF, PR, and MLP all exhibit R‐squared values close to 1 (0.980, 0.981, 0.968, and 0.980, respectively), with no statistically significant differences among them, indicating excellent performance. However, the LSTM model shows a slightly lower R‐squared value of 0.895. Despite this, it remains close to 1, suggesting that all the trained models demonstrate sufficient overall performance.

### 2.7 Computation Times Required for the Traditional Machine Learning Algorithms

Optimization of computation time is an essential factor that directly impacts a model's performance and the efficiency of the development process, cost‐effectiveness, and practical applicability. DL models often require longer training times to fully leverage the depth and complexity of neural networks compared to traditional ML models (Figure 6e; **Table** **4**). This extended training time can lead to significant performance improvements, particularly for tasks requiring high‐level feature extraction. As shown in Figure 6e, the MLP model took 4.296 sec for training and 0.218 sec for testing, while the LSTM model required 9.349 sec for training and 0.350 sec for testing. Longer training times allow the model to converge more effectively, reducing the loss function and improving accuracy. However, excessively long training times can result in overfitting, where the model performs well on the training data but poorly on unseen data. These risks can be mitigated using regularization techniques and early stopping.^[^ ^40^ ^]^

**Table 4:** Computation times of three traditional machine learning algorithms and two deep learning algorithms were measured using the dataset to evaluate their performance.

Algorithms	Training time (ms)	Testing time (ms)
Decision tree	0.005 ± 0.002	0.005 ± 0.001
Random forest	0.020 ± 0.001	0.005 ± 0.001
Polynomial regression	0.345 ± 0.005	0.011 ± 0.001
Multilayer perception	4.296 ± 0.111	0.218 ± 0.026
Long short‐term memory	9.349 ± 0.202	0.350 ± 0.020

Additionally, computation time is directly related to cost. While longer training times can improve performance, they also increase computational expenses. Balancing training duration with available resources is important for practical applications. Increased training time can enhance model performance by enabling better convergence, thorough hyperparameter optimization and robust validation. However, it is essential to balance these benefits with the risk of overfitting and real‐world constraints on computational resources.^[^ ^40^ ^]^

### 2.8 Development of User‐Interface Web Application

Building on the algorithm's demonstrated high prediction accuracy, the predicted droplet volumes closely align with the measured volumes, indicating the model's effectiveness in accurately capturing the characteristics of the droplets. This consistency between predicted and actual values highlights the algorithm's reliability in practical applications (Figure 6f,j). In addition, a front‐end web application was developed based on these models, as illustrated in Figure 6k. This user‐friendly interface leverages the high‐performance predictive model to provide real‐time, accurate estimations of droplet volumes. The web interface is divided into two main sections: parameters and predictions. The parameters section includes viscosity, nozzle size, dispensing time, printing pressure, and cell concentration. By adjusting these parameters, users can obtain predicted droplet volumes before cellular droplet bioprinting, facilitating a faster, easier, and more precise acquisition of the desired cellular droplets. The prediction section presents results in three columns: algorithm, volume, and accuracy. The algorithm column specifies the type of algorithm used, the volume column shows the predicted droplet volume, and the accuracy column indicates the precision of each algorithm. Data for droplet size prediction was gathered using computer vision techniques and equations, with the results compiled in CSV format (Figure 6l). This CSV data was then used as the training dataset for the ML algorithms, which were implemented using Python.

To provide access to the trained ML algorithms as a web service, we developed an application programming interface (API) server. The web application communicates with this API server, enabling users to interact with the prediction models through a user‐friendly web interface. Through this interface, users can input parameters, submit requests to the server, and receive predictions for droplet sizes. This integrated system allows researchers to efficiently predict and optimize cellular droplet volumes before bioprinting, significantly enhancing the precision and effectiveness of the bioprinting process.

### 2.9 Assessment of Cellular Viability and Proliferative Capacity in 3D Bioprinted Constructs

**Figure 7:**
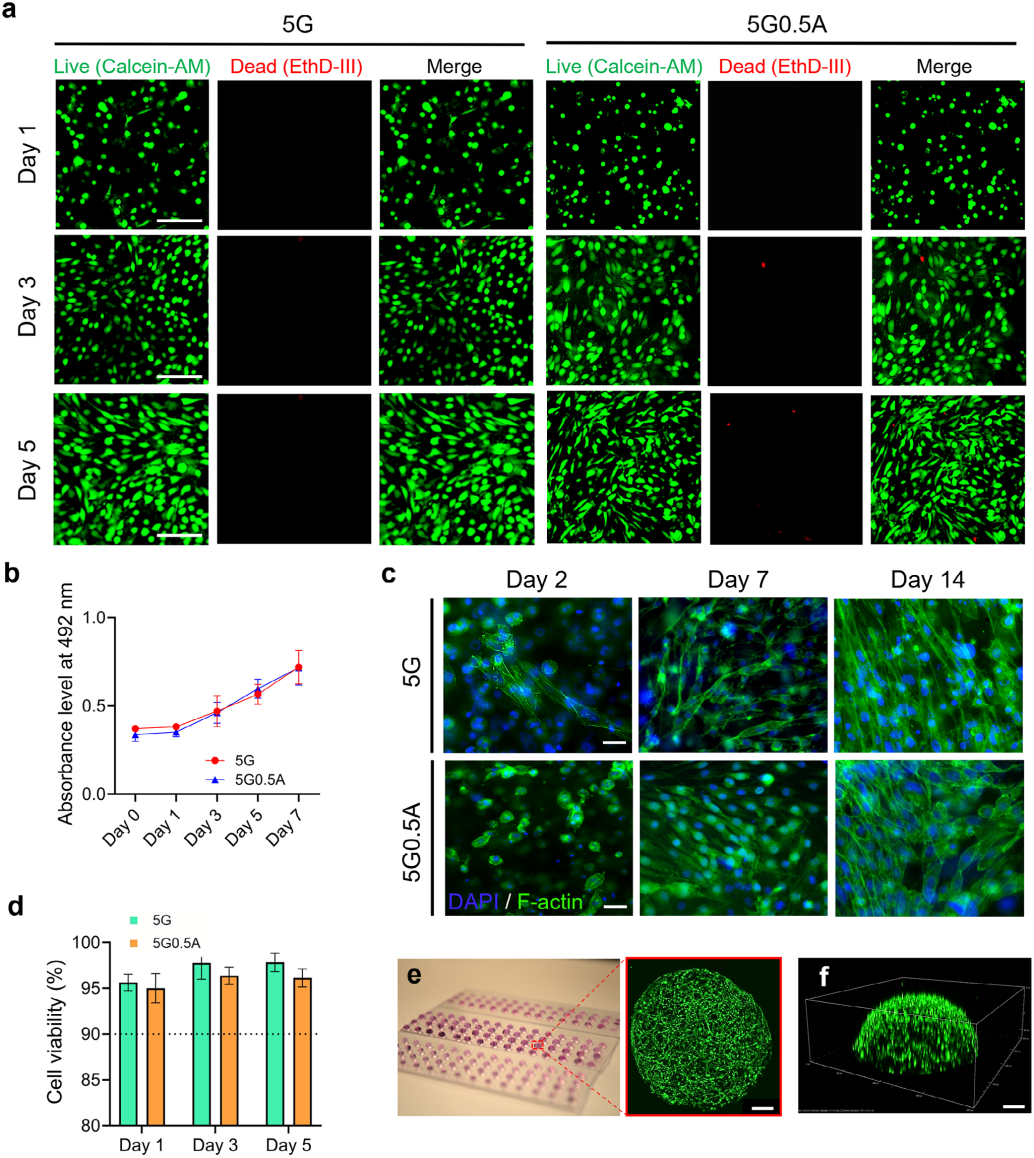
a) Representative images showing live (green) and dead (red) NIH 3T3 fibroblast cells at days 1, 3, and 5. Scale bar = 100 µm. b) Cell proliferation rate from day 0 to 7. c) 3T3 cells embedded in GelMA‐alginate hydrogels were observed over two weeks under 5G and 5G0.5A conditions. Cell nuclei were stained with DAPI (blue) to represent the original cells, while the cytoskeleton was visualized using phalloidin (green). Scale bar = 50 µm. d) Cell viability is determined by counting live and dead cell populations on days 1, 3, and 5. e) Maximum intensity projection images through 1 mm thickness of bioprinted GFP‐3T3 cell encapsulated in 5G2A bioink. Scale bar = 200 µm. f) 3D confocal imaging of GFP‐3T3 cells encapsulated in 5G2A bioink. Scale bar = 200 µm.

Maintaining cell viability while minimizing shear stress‐induced damage at the nozzle is critical. As shown in **Figure** **7**a, GFP‐3T3 cells encapsulated in 5G and 5G0.5A hydrogels exhibited numerous cell elongations, indicating favorable cell‐matrix interactions. Proliferation assays conducted up to day 7 revealed a steady increase in cell numbers (Figure 7b). In contrast, cells in the 5G material initially showed higher proliferation rates, and by day 3, both 5G and 5G0.5A compositions supported comparable proliferation kinetics. Cellular morphology and nuclear integrity were further confirmed through phalloidin and DAPI staining, respectively (Figure 7c). Quantitative analysis of cell viability from day 1 to 5 consistently demonstrated viability rates exceeding 90% (Figure 7d).

To observe the overall distribution of cell growth within the 3D droplet, GFP‐3T3 cells were encapsulated in droplets with a thickness ranging from 500 to 1 mm. These droplets were then bioprinted and cultured in an appropriate medium. The time‐lapse video captured on day 3 demonstrates cell elongation and proliferation from the bottom to the top area of the droplet (Movie S6, Supporting Information). On day 3, after bioprinting, confocal imaging of GFP‐3T3 cells encapsulated in the bioink scaffold was performed. Figure 7e presents maximum intensity projection images of a droplet ≈1 mm thick, captured at 10X magnification using 9 tiled images. Figure 7f displays a 3D view of the same sample, illustrating the complete elongation of the cells by day 3. These results demonstrate both the hydrogel formulations' biocompatibility and the cell‐preserving capabilities of our bioprinting system.

## 3 Discussion

Droplet bioprinting of cell aggregates and organoids at the microliter scale presents significant challenges, necessitating the optimization of bioprinting parameters prior to printing.^[^ ^41^ ^]^ Maintaining precise control during bioprinting and ensuring high repeatability and stability of the resulting cell‐laden constructs remain challenges that need to be addressed.^[^ ^42^ ^]^ To address these challenges, we proposed an innovative strategy for optimizing bioprinting parameters by utilizing ML technologies. One of the primary challenges in integrating ML with bioprinting is the collection of large, high‐quality datasets. To address this, we developed a cost‐effective assistive system, incorporating a linear stage and USB camera, to facilitate large‐scale data collection and ML optimization. Additionally, a web‐based user interface was created to enable users, even those with limited bioprinting experience, to easily optimize printing parameters using the ML model. This standalone, versatile system can be integrated into existing bioprinters, enhancing accessibility, functionality, and adaptability for a wider range of researchers.

The ML model in this study specifically tackles the issues of optimizing droplet volume consistency and cell viability, which are critical for high‐throughput 3D bioprinting processes. Traditional methods rely heavily on manual adjustments and empirical testing, which are both time‐consuming and imprecise. By predicting droplet volumes based on key bioprinting parameters, our model enables real‐time parameter optimization, significantly improving precision, reproducibility, and efficiency in bioprinting.

The predictive performance was evaluated based on two criteria: droplet volume prediction accuracy and computation time. We recognize the importance of clearly articulating the rationale for comparing multiple models. The practical significance of leveraging DL models, such as MLP and LSTM, over traditional regression methods, such as DT, RF, and PR, lies in their ability to capture complex, nonlinear parameter relationships with superior prediction accuracy. Additionally, these comparisons provide valuable insights into selecting the most appropriate predictive models based on specific computational constraints and accuracy requirements. Computation time was considered an important metric to assess the practicality and scalability of the methods. While this task does not require real‐time analysis or involve excessively long training periods, computation time becomes a critical factor when scaling up to larger datasets or more complex models, which are often encountered in real‐world bioprinting applications. Moreover, researchers operate with varying computational resources, and understanding the balance between model performance and computational efficiency can help guide the selection of appropriate algorithms based on specific constraints and requirements. By presenting this comparative analysis, we aim to guide researchers in making informed decisions that align with their unique experimental needs and resource availability.

For the first criterion, the MLP algorithm outperformed other models. It achieved the lowest MAE, MSE, and RMSE values, indicating high precision in droplet volume predictions. Additionally, the MLP's R‐squared value was close to 1, reflecting a strong correlation between the predicted and actual droplet volumes. This underscores the MLP's robustness in handling the complexities of droplet bioprinting parameter predictions.

Regarding computation time, the DT algorithm exhibited the shortest training and testing times due to its relatively simpler architecture compared to DL.^[^ ^43^ ^]^ This makes it an attractive option for scenarios where rapid computation is essential. Nonetheless, the MLP also demonstrated efficient computational performance, with training times under 4 sec and testing times under 2 sec suggesting that it can be effectively used in real‐time bioprinting applications without significant delays.

Overall, this study highlights the potential of ML in optimizing bioprinting parameters for more efficient bioprinting. The MLP, with its superior prediction accuracy and reasonable computation time, offers a balanced approach to enhancing bioprinting precision. Meanwhile, the DT algorithm provides an alternative when computation speed is the priority. These insights contribute to the advancement of bioprinting technologies, ultimately aiding in the creation of high‐quality, reproducible bioprinted constructs for applications in tissue engineering and precision medicine.

Among bioprinting parameters, bioink composition plays a critical role in extrusion‐based bioprinting.^[^ ^44^ ^]^ Low concentrations of hydrogel bioink often result in inadequate mechanical stability and printability, complicating the creation of constructs with microliter‐sized droplet volumes. Based on the mechanical characterization, 5G0.5A and 5G1A were identified as the most suitable candidates for droplet bioprinting and were subsequently selected for further optimization.

Samples with a high concentration of methacryloyl groups demonstrated rapid initiation of the photocrosslinking reaction, as evidenced by the immediate increase in storage modulus. This behavior is characteristic of highly functionalized GelMA, where the abundance of crosslinkable groups facilitates rapid network formation.^[^ ^45^ ^]^ Increasing alginate concentration correlated with a slight decrease in the final storage modulus. This phenomenon can be attributed to the interference of alginate with the GelMA photocrosslinking process. Alginate molecules may physically impede the interaction between methacryloyl groups, resulting in a less densely crosslinked network and, consequently, lower elastic properties.

Regarding the 3D bioprinter, we customized it to enhance the bioprinting process's controllability and to collect a high‐throughput image dataset for ML optimization training. The presented bioprinting system was also designed with scalability in mind, enabling high‐throughput organoid production by accommodating several multi‐well plates within the print bed for simultaneous printing. This configuration supports mass production workflows and facilitates efficient scaling of organoid manufacturing processes. This bioprinter enabled the controlled bioprinting of microliter‐sized cell‐laden droplet constructs. To ensure a consistent cell number in each droplet, we installed a cell stirring system within the syringe, the effectiveness of which was verified by measuring cell numbers at three different time points during the bioprinting process. Additionally, bioprinting was conducted on a hydrophobic glass slide surface to enhance the clarity and repeatability of the droplet image edges. This approach not only increased the contact angles of the droplets to over 90^°^ by making the surface hydrophobic but also facilitated bonding between the hydrogel precursor and the glass. This ensures suitability for long‐term culture and keeps the constructs securely attached for extended periods.^[^ ^43^, ^46^ ^]^

The high‐throughput images collected were transferred to a computer for further analysis. Three distinct image processing techniques were applied to these images. By employing multiple image processing methods, we mitigated potential inaccuracies inherent in a single technique, thereby enhancing the robustness of our volume measurements. This comprehensive image analysis streamlines not only provided a high degree of control over the bioprinting process but also contributed to the optimization and standardization of our bioprinting protocols.

The diverse droplet volumes produced by adjusting bioprinting parameters were input into three traditional ML algorithms and two DL algorithms for training and testing. Before this, hyperparameter optimization was performed for each algorithm to identify the configuration that yielded the best predictive performance. By employing hyperparameter optimization, each algorithm was tuned to ensure the highest predictive accuracy for droplet volume outcomes. In addition, this process enhanced the model's generalization performance, reducing the risk of overfitting. This rigorous approach allowed us to systematically evaluate and improve the performance of our bioprinting process. The combination of multiple algorithms provided a comprehensive understanding of the parameters influencing cell‐laden droplet formation, facilitating the development of robust predictive models. These models are crucial for real‐time optimization of bioprinting parameters, thereby improving the consistency and quality of bioprinted constructs.

ML offers a faster and more accurate approach to maintaining consistency in bioprinting compared to traditional methods. To ensure the long‐term stability and consistency of the bioprinting process, the ML model's prediction accuracy is evaluated weekly. This regular validation demonstrates the robustness of the system over time. In future work, a calibration system will be developed and integrated into the existing web‐based user interface to further enhance precision and usability. These contributions significantly enhance the precision and scalability of organoid manufacturing, providing a robust foundation for future developments in tissue engineering and precision medicine.

## 4 Conclusion

We present a highly efficient bioprinting platform capable of generating high‐throughput cellular droplets, integrated with an AI‐based optimization system. This innovative approach leverages the power of AI to significantly enhance the bioprinting process, moving beyond the traditionally time‐consuming trial‐and‐error methods required to achieve optimal droplet volumes for organoids and cellular droplet bioprinting. Droplet volumes were calculated by analyzing droplet diameters and contact angles from high‐resolution images, ensuring exceptional accuracy in the predictions.

By utilizing three modified traditional ML algorithms and two DL algorithms, we have developed predictive models capable of accurately determining cell‐laden droplet volumes based on a comprehensive set of bioprinting parameters. These models show exceptional proficiency in capturing the complex relationships between input parameters and droplet volume outcomes. The top‐performing model achieves remarkable prediction accuracy and demonstrates impressively short training and testing times, representing a significant advancement in the efficiency and effectiveness of bioprinting applications.

We employed four regression model‐based evaluation metrics to assess the results. Each metric provides valuable insights depending on the study's objectives and the nature of the data. The RMSE evaluation index identified MLP and LSTM as the most accurate regression models. In the R‐squared evaluation index, which reflects the volatility of the data, DT, RF, PR, and MLP all produced excellent results with values ​​above 0.95. Regarding computational time, the traditional ML algorithm demonstrated significantly faster speeds compared to the DL algorithm.

This innovative approach streamlines the optimization process, significantly reducing bioink wastage and time, thereby addressing a major challenge in the field. By combining cutting‐edge ML techniques with advanced bioprinting technology, this approach opens new frontiers in tissue engineering and precision medicine. This innovation holds the potential to accelerate research, enhance therapeutic outcomes, and ultimately contribute to improved health and well‐being for countless individuals worldwide.

## 5 Experimental Section

### Synthesis and Preparation of Bioinks

GelMA‐based bioink had been demonstrated to enhance cell viability and improve printing quality during the extrusion‐based bioprinting process.^[^ ^47^ ^]^ The synthesis of GelMA was conducted according to a previously reported protocol.^[^ ^47^ ^]^ In summary, the procedure involved dissolving 5 g of porcine skin‐derived powdered gelatin (Type A, Bloom strength 300, Sigma‐Aldrich, St. Louis, MO, USA) in 50 mL of distilled water. Upon achieving complete dissolution at 50 °C, 10 mL of glycidyl methacrylate (Sigma‐Aldrich, St. Louis, MO, USA) was gradually added dropwise into the dissolved gelatin solution. The resultant solution was maintained for 12 h at 50 °C with continuous stirring at 750 RPM. The solution was then transferred into a dialysis tube with a molecular weight cutoff of 12–14 kDa (Fisher Scientific, Waltham, MA, USA). Over the subsequent three days, the water in the dialysis tube tank was replaced twice daily. Following this period, the solution was frozen at −80 °C for 24 h, followed by lyophilization at −84 °C for three days to obtain dried GelMA foam. The synthesized GelMA was stored at −20 °C for future use.

To prepare the GelMA‐alginate hydrogel, a stock solution of 5% (w/v) GelMA in PBS (VWR, Radnor, PA, USA) containing 0.2% lithium phenyl‐2,4,6‐trimethylbenzoylphosphinate (LAP) photoinitiator (Sigma‐Aldrich, St. Louis, MO, USA) was prepared (referred to as stock 1). The presence of alginate supported the stability of the hydrogel precursor during bioprinting.^[^ ^48^ ^]^ The increased viscosity of the hydrogel precursor, due to the addition of alginate, was particularly beneficial when bioprinting very small droplets ranging from 0.1 to 2 µL. Alginate solutions with concentrations of 0.5 and 1% (w/v) were prepared in stock 1, and vigorous agitation was applied for 3 h to ensure complete dissolution.

The GFP‐tagged NIH 3T3 fibroblast cells (ATCC, Manassas, VA, USA) were utilized for bioprinting optimization and in vitro experiments. Cells were maintained in Dulbecco's Modified Eagle Medium (VWR, Radnor, PA, USA) supplemented with 10% fetal bovine serum (VWR, Radnor, PA, USA) and 1% penicillin‐streptomycin (VWR, Radnor, PA, USA). Culture conditions were maintained at 37 °C and 5% CO_2_, with the growth medium, replaced every 48 h. Upon reaching 90% confluency, cells were harvested by incubation in trypsin‐EDTA solution (VWR, Radnor, PA, USA) for 5 min, followed by centrifugation for 3 min at 1500 RPM. For optimization and in vitro characterization, the harvested cells were suspended in two bioink formulations: 5G0.5A and 5G1A. The hydrogel characterization also included 5G and 5G2A.

### Bioink Characterization

Rheological property characterization was conducted using a rheometer (MCR 302e, Anton Paar, Graz, Austria) equipped with a Peltier plate for the 5G0.5A and 5G01A materials used in the ML training and 5G and 5G2A as control groups. A stainless steel parallel plate with a diameter of 25 mm and a gap distance of 0.5 mm at room temperature was utilized for all experiments. Rheological tests assessed the flow characteristics and viscoelastic behavior pertinent to the bioprinting process.

To verify the photocrosslinking kinetics, a transparent glass plate was integrated into the experimental setup, allowing for the illumination of the hydrogel with a 405 nm light source from beneath the 25 mm parallel plate geometry. The initial 60 sec period with the light off established the baseline viscoelastic properties of the uncrosslinked materials. Upon light activation at 60 sec, the evolution of storage modulus (G') and loss modulus (G'') provided insight into the hydrogels' crosslinking kinetics and final mechanical properties. Oscillatory measurements were conducted using a 1% shear strain and a frequency of 1 Hz. Concurrently, both the storage and loss moduli for the photocrosslinking bioink were recorded.

The transparency of samples printed in the shape of a disc was investigated to enhance the efficiency of the light permeability during the photocrosslinking process. Additionally, the transparent sample facilitated the optical observation of cells and their surrounding environment within the hydrogel. To assess the transparency, crosslinked samples were prepared in the form of discs, each with a diameter of 8 mm and a height of 6 mm, and placed on a sheet of paper bearing the university logo.

To determine the viscosity of 5G0.5A and 5G1A materials, the zero‐shear viscosity was measured by setting the shear rate to 1 s^−1^. The mechanical properties of photocrosslinked hydrogel were evaluated by measuring the compressive elastic modulus, which reflected the stiffness of its microstructure. Hydrogel precursors were dispensed into molds with a diameter of 8.5 mm and a height of 3.5 mm, with each disc receiving 300 µL. After crosslinking with 405 nm light for 1 min, the samples were immediately tested for compressive modulus. A vertical‐axis micromechanical testing machine (ESM 301L, Mark‐10, USA) equipped with a custom‐made flat‐ended rigid cylinder (12.5 mm in diameter) was used to perform compression tests. This equipment recorded force‐displacement measurements during indentation. Force‐displacement plots were generated by subjecting the hydrogel surface to a 50% displacement at a 10 mm min^−1^ speed. Utilizing the initial dimensions of the hydrogel samples and custom MATLAB code, the compression modulus was calculated based on the slope within the initial 10% strain region, known as the linear region.

Evaluating the water absorption capacity in crosslinked hydrogel samples required assessing their swelling ratio. Initially, 300 µL of pre‐polymer solutions were loaded into molds with an 8 mm diameter. These solutions were then crosslinked under 405 nm light for 1 min. After crosslinking, the hydrogels were immersed in PBS and maintained at 37 °C for 24 h to ensure complete hydration. Upon achieving equilibrium swelling, the weight of each sample in its hydrated state (W_w_) was accurately recorded. Subsequently, the hydrated hydrogel samples were frozen in a −80 °C freezer and subjected to lyophilization for three days to determine their dry weight (W_d_). Finally, Equation (2) was used to calculate the swelling ratio, with the entire process being repeated three times for consistency.

(2) $$\mathrm{Swelling}\;\mathrm{ratio}=\frac{\mathrm{Ww}-\mathrm{Wd}}{\mathrm{Wd}}\times 100$$

### Development of a 3D Bioprinting Platform Specialized in Machine Learning‐Driven Droplet Optimization

A fully customizable 3D bioprinting system was developed by incorporating additional components to facilitate the high‐throughput dataset generation essential for training ML algorithms. The bioprinting process was controlled by pre‐defined G‐code instructions, which directed the bioink dispensing and printed bed movement based on droplet volume and spacing requirements. This ensured precise deposition and prevented droplet merging. An additional advantageous feature of this system was its micro‐dropletization capability, enabling the production of droplets with an average diameter of ≈100 µm. This functionality was particularly valuable for constructing single‐cell arrays, allowing for the precise delivery of individual cells to specific locations. The system's efficiency was demonstrated by its ability to rapidly and accurately bioprint 60 droplets, each with a 4 µL volume, on a coated hydrophobic glass slide (VWR, Radnor, PA, USA) (Movie S7, Supporting Information).

Accurate measurement of droplet volume required the determination of both diameter and contact angles. The diameter was determined using a calibrated pixel‐to‐length ratio method, which defined the length represented by each pixel. This method provided a robust framework for precise diameter determination and ensured accuracy in the quantitative analysis of droplet dimensions.

### Droplet Volume Calculation and Image Processing

The calculation of contact angles was performed through image processing. In the methodology proposed in this work, the raw images underwent a comprehensive series of three distinct image processing stages. First, the recorded multi‐channel images were converted into grayscale, thereby optimizing memory utilization by integrating the three red, green, and blue channels into a single‐channel grayscale matrix. With a specific focus on contrast and brightness parameters, this grayscale conversion facilitated the extraction of clearer droplet features, enhancing visual discernibility. Second, the process involved Canny edge detection processing. Employing a Gaussian filter mitigated noise within the image, resulting in a more precise delineation of droplet edges. Subsequently, the images were converted to a binary format. Binary images, characterized by pixels with only two values, streamlined subsequent image‐processing tasks. The relevant area was represented in black to accentuate the droplet pattern within the image, while the remaining portions were depicted in white. This deliberate binary representation effectively highlighted the droplet within the image. Finally, all processed images, encompassing grayscale, Canny edge detection, and binary representations, were combined and saved as the final combined image.

To validate the accuracy of the developed image processing algorithm, droplet volumes from a subset of images were compared with those calculated using ImageJ, a widely used traditional method for image analysis. The analysis in ImageJ employed the contact angle plugin to manually define key points, including the two endpoints where the droplet met the substrate and five additional points along the droplet boundary. These points were used to measure the droplet area, which was subsequently applied to calculate the volume under the assumption of a spherical shape.

While ImageJ provided reliable measurements, its manual nature made it labor‐intensive and inefficient for processing large batches of images. To address this limitation, a custom image processing algorithm was developed in this study to automatically and efficiently manage large datasets. This automated pipeline not only provided a scalable alternative for droplet volume measurement but also served as a reference point for comparing the performance and accuracy of manual versus automated methods.

### Bioprinting Preparations and Parameters—

The silane‐coated glass substrate traveled along the x‐axis to accommodate multiple cell‐laden droplets at uniform spacing. These deposited droplets were subsequently exposed to a 405 nm lamp to polymerize the photo‐crosslinkable biomaterials. Printing parameters and corresponding droplet volumes served as input datasets for training the ML and DL algorithms (Table 4). Two bioink formulations with different viscosities were utilized: 5G0.5A (10.03 mPa·s) and 5G1A (24.66 mPa·s). The bioprinting process incorporated three conical needles with varying inner diameters of dispensing nozzles. Printing parameters were systematically adjusted, with printing times set at 0.05, 0.1, and 0.15 sec and printing pressures set to 1.5 and 2 psi, respectively. The cell‐laden biomaterials were prepared in 3 mL volumes, with two different cell concentrations: 2.8 × 10^6^ and 5.6 × 10^6^ cells/3 mL.

The parameter weights presented in Figure 3e,f were derived using the feature importance metrics of the ML models. For the DT and RF models, the Random Forest Regressor class from the scikit‐learn library and its feature importances attribute were utilized. Scikit‐learn library, a popular Python library for ML, provided a comprehensive suite of tools for tasks. The feature importances attribute quantified each feature's contribution to reducing prediction error, such as MSE. This was calculated by summing the reduction in impurity (e.g., Gini impurity or MSE) at each DT node where the feature was used to split data. In Random Forest Regressor, these values were averaged across all trees and normalized so that the total importance equals one. This metric provided an interpretable measure of variable importance, highlighting the parameters with the most significant impact on predictions. It enabled researchers to identify key features driving the model's predictions and prioritize them for further analysis.

### Bioprinting Preparations and Parameters—Traditional Machine Learning (ML)

In this study, supervised learning, a fundamental paradigm in ML that utilized labeled datasets, was implemented. Within this framework, the research focused on regression models, as the primary objective was to develop models capable of predicting continuous output values based on corresponding input data.

### Bioprinting Preparations and Parameters—Decision Tree (DT)

The concept of a DT was based on a structure resembling a combination of trees and branches, characterized by its ability to operate efficiently even on complex datasets.^[^ ^49^ ^]^ The DT algorithm partitions data based on specific criteria or questions, with its effectiveness relying on generating optimized questions. It was based on the classification and regression tree algorithm, which began at the root node, representing the initial depth, and formed a binary tree by dividing the data into two regions for each node branch.^[^ ^50^, ^51^ ^]^ Each branch contained a rule node at the midpoint and a leaf node at the end, representing the outcome. While increasing the number of rule nodes improved predictive learning, it also added complexity, which could lead to overfitting. We trained a DT algorithm using 10‐fold cross‐validation with max_depth values ranging from 1 to 19. For each depth, the model was trained on 10 different training and test datasets. We recorded performance metrics, including train_mae (mean absolute error for the training dataset), val_mae (mean absolute error for the validation dataset), train_rmse (root mean square error for the training dataset), val_rmse (root mean square error for the validation dataset), train_r2 (R‐squared for the training dataset), val_r2 (R‐squared for the validation dataset), train_mse (mean squared error for the training dataset), and val_mse (mean squared error for the validation dataset). Analysis of these results revealed that a max_depth of 7 achieved the optimal balance, effectively avoiding overfitting.

DT was utilized in both classification and regression tasks. Three key metrics: Gini index, entropy, and classification error were used to measure data impurity in a binary DT.^[^ ^50^ ^]^ A lower Gini index indicated greater data uniformity, and the method that produced the highest information gain was selected to derive results. Impurity acted as a classification standard, enabling the creation of branches with lower impurity by clearly distinguishing between classes and grouping identical objects. In contrast, DT designed for regression models measured impurity using MSE.^[^ ^52^ ^]^

### Bioprinting Preparations and Parameters—Random Forest (RF)

In RF, five primary types of hyperparameters (e.g., n_estimators, criterion, max_depth, min_samples_split, and min_samples_leaf) were critical (Table S3, Supporting Information). Hyperparameter tuning was essential for developing an optimal training model.^[^ ^53^ ^]^ To determine the optimal number of estimators (n_estimators), we experimented with values ranging from 1 to 19. This was achieved using 10‐fold cross‐validation, where the data was split into ten distinct training and test datasets for each number of estimators. This method enabled a thorough evaluation of the model's performance and helped identify the most suitable number of trees in the forest while minimizing the risk of overfitting.

By comparing performance metrics across different numbers of estimators, we found that ten estimators provided the best balance between training and validation performance. At this configuration, the model exhibited robust performance on both datasets, effectively capturing the underlying patterns in the data without overfitting. Thus, we concluded that 10 estimators represent the optimal value for our RF model, maximizing predictive performance on new data while maintaining a balance between complexity and generalization.

While the optimal conditions for these five hyperparameters could be manually determined, this study utilized an automated training method with optimized hyperparameter combinations. RandomizedSearchCV selected a specified number of random combinations from a predefined hyperparameter search space and outputted the combination with the best score.^[^ ^54^ ^]^ In contrast, GridSearchCV systematically explored all possible combinations of hyperparameter values. In this study, GridSearchCV was chosen due to its superior performance.^[^ ^55^ ^]^

### Bioprinting Preparations and Parameters—Polynomial Regression (PR)

In contrast to linear regression (Equation 3), which established a linear relationship between a single independent variable and a dependent variable, and multiple linear regression (Equation 4), which involved multiple independent variables and a single dependent variable, PR utilized a polynomial function.^[^ ^56^ ^]^ This approach captured non‐linear relationships between independent and dependent variables, enabling the model to identify intricate patterns in the data and improved predictive accuracy. In PR, the hyperparameter “degree of polynomial” determined the highest power of the polynomial used in the model (Equation 5).^[^ ^57^, ^58^ ^]^

(3) y=mx+b

(4) $$y=b+m_{1}x_{1}+m_{2}x_{2}+\cdots +m_{n}x_{n}$$

(5) $$y=b+m_{1}x+m_{2}x^{2}+\cdots +m_{n}x^{n}$$

In analyzing data with a PR model, we evaluated polynomial degrees ranging from 1 to 9. Performance improved on the training dataset as the degree increased but deteriorated on the validation dataset beyond a certain point, indicating overfitting. By comparing performance metrics, it was identified that a polynomial degree of 7 provided the best balance between training and validation performance. This degree effectively captured data patterns without overfitting. Consequently, it was concluded that a polynomial degree of 7 was optimal for maximizing predictive performance while maintaining a balance between model complexity and generalization.

### Bioprinting Preparations and Parameters—Deep Learning (DL)

DL was a subset of ML that utilized multilayer neural networks composed of an input layer, one or more hidden layers, and an output layer. These networks, often referred to as artificial neural networks, form the foundation of DL.^[^ ^59^, ^60^ ^]^

### Bioprinting Preparations and Parameters—Multilayer Perception (MLP)

To determine the optimal number of epochs for our MLP model, we tested epoch counts ranging from 10 to 100 in increments of ten using 10‐fold cross‐validation. This approach involved splitting the data into ten training and test sets for each epoch count, allowing for a comprehensive evaluation while minimizing overfitting. Performance on the training dataset improved with more epochs, but beyond a certain point, validation performance stabilized, indicating effective learning without significant overfitting. By comparing metrics across epoch counts, it was determined that 100 epochs provided the best balance between training and validation performance. Therefore, 100 epochs were deemed optimal for maximizing predictive performance while maintaining a balance between complexity and generalization.

### Bioprinting Preparations and Parameters—Long Short‐Term Memory (LSTM)

LSTM, a type of recurrent neural network, was widely used in DL for processing sequential or time‐dependent data.^[^ ^61^ ^]^ Unlike feedforward neural networks, such as MLP, which were designed for non‐sequential data, LSTM excelled at identifying patterns within sequences. LSTM was employed in this study to model the sequential dependencies inherent in the bioprinting process, where each step's outcome was influenced by preceding parameters and outputs. Bioprinting involved the continuous interaction of multiple factors, including printhead movement, printing speed, pressure, ambient temperature, and environmental humidity, all of which collectively impacted the final print quality. Given that prolonged printing sessions could amplify these temporal dependencies, a temporal guideline was established to assist researchers in optimizing bioprinting conditions. Additionally, to further minimize variations caused by environmental fluctuations, a fully controlled bioprinting environment with stable temperature and humidity regulation was planned for development.

In analyzing data with an LSTM model, the optimal number of epochs were determined by testing values ranging from 10 to 100 in increments of ten. Using 10‐fold cross‐validation, the data were split into ten training and test sets for each epoch count, enabling a comprehensive evaluation of model performance while mitigating overfitting. It was observed that while performance on the training dataset improved with increasing epochs, it began to decline on the validation dataset beyond a certain point, indicating overfitting. By comparing performance metrics, it was determined that 100 epochs provided the best balance between training and validation performance. Thus, it was concluded that 100 epochs were optimal for our LSTM model, maximizing predictive performance while maintaining a balance between complexity and generalization.

### Bioprinting Preparations and Parameters—Analysis of the Dataset

To ensure a balanced dataset for training the ML models, viscosity was used as the basis for dividing the dataset. The dataset was adjusted to include an equal number of data points for the two bioink compositions: 5G0.5A bioink (viscosity: 10.03 mPa·s) and 5G1A bioink (viscosity: 24.66 mPa·s). This approach minimized potential biases caused by unequal representation of bioink compositions, thereby enhancing the model's robustness and generalizability.

Each model underwent a dataset partitioning process, where the entire dataset, consisting of 1758 instances, was randomly partitioned into two sets: a training set (70% of the data) and a testing set (30% of the data).^[^ ^62^ ^]^ Within the training set, 10‐fold cross‐validation was performed by splitting the data into ten mutually exclusive subsets. This approach systematically assessed model performance and enhanced generalization capabilities.^[^ ^63^ ^]^ During cross‐validation, the model training and evaluation processes were repeated ten times, each time using a different subset for testing while the remaining subsets were used for training. This iterative procedure produced ten individual performance scores, which were averaged to provide a final, comprehensive evaluation of model performance.

The ultimate performance metric was obtained from the results of the testing set. Image preprocessing, data analysis, and the training of ML and DL models were conducted using Python version 3.12.1, with TensorFlow and scikit‐learn libraries (available on GitHub). Detailed information about the models and tuning parameters was provided in the following sections.

### Bioprinting Preparations and Parameters—Optimization of the Hyperparameters

Hyperparameters were configuration settings that control and adjust a model's learning process before training.^[^ ^64^ ^]^ Tuning these hyperparameters was crucial, as it impacted the model's structure, learning procedure, and optimization method. In this study, distinct hyperparameters were tuned for each algorithm. For the DT algorithm, the max_depth hyperparameter was optimized to determine the maximum tree depth.^[^ ^65^ ^]^ RF models were fine‐tuned using five specific hyperparameters (Table S3, Supporting Information). In the case of PR, the polynomial degree hyperparameter was manually adjusted to identify the optimal degree of the polynomial function. For DL models, such as MLP and LSTM, hyperparameters like learning rate, number of epochs, and batch size were predefined and manually tuned to optimize model performance. During training, the model iteratively updated its parameters, such as weights and biases, to minimize the loss function and improve predictive accuracy. In this study, hyperparameters were systematically tuned using grid search, and their impact on model performance was evaluated using 10‐fold cross‐validation.

### Bioprinting Preparations and Parameters—Evaluation Metrics for Comparative Analysis Between Machine Learning Algorithms

The regression‐based traditional ML and DL models presented in this study were compared and analyzed using four evaluation criteria. MAE measured the absolute difference between predicted and actual values.^[^ ^39^ ^]^ A lower MAE indicated that the model's predictions were closer to the actual values. MSE measured the squared difference between predicted and actual values. MSE was calculated by squaring each prediction error, summing these squared values, and dividing by the total number of data points to calculate the mean. It always produced non‐negative values and assigned greater weights to larger errors. A lower MSE meant that the model's predictions aligned more closely with the actual values. The RMSE was derived by taking the square root of the MSE. RMSE represented the average magnitude of prediction errors, expressed in the same units as the target variable. A smaller RMSE indicated that the model's predictions were closer to the actual values. R‐squared was a metric used to assess the goodness of fit in a regression model, indicating how well the model explained the variability of the data on a scale from 0 to 1.^[^ ^39^ ^]^ A value closer to 1 signified a well‐trained model, demonstrating high suitability to the given data. The higher the R‐squared value, the better the model was considered to explain the data. However, R‐squared values approaching 1 could sometimes indicated overfitting, so it was advisable to use additional evaluation metrics alongside R‐squared to ensure a balanced assessment.

### Statistical Analysis

To evaluate the performance differences among three traditional ML and two DL algorithms and other relevant factors, one‐way and two‐way ANOVA were conducted using Minitab 20 (Minitab, LLC., PA, USA). The null hypothesis assumed that all algorithms exhibited equivalent performance. Rejection of the null hypothesis indicated statistically significant differences in prediction performance among the groups. The ANOVA analysis was followed by Tukey's honestly significant difference (HSD) test as a post hoc analysis. Tukey's HSD test identified specific algorithms that differed significantly in their mean performance. The evaluation framework utilized a 10‐fold cross‐validation approach, requiring ten iterations of the analysis. This robust methodology ensured the reliability and generalizability of performance comparisons across the ML algorithms under investigation.

## Conflict of Interest

The authors declare no conflict of interest.

## Author Contributions

The project and concept were conceived by J.S. K.K. and J.S. conducted all of the experiments and wrote the final manuscript with contributions from all authors. M.K. contributed to data processing and worked on developing the algorithms and web user interface. K.H. and H.K. assisted in modifying the 3D bioprinting system. All authors participated in this research, discussed the results, and provided comments on the manuscript.

## Supporting information



Supporting Information

Supplemental Movie 1

Supplemental Movie 2

Supplemental Movie 3

Supplemental Movie 4

Supplemental Movie 5

Supplemental Movie 6

Supplemental Movie 7

## Data Availability

The data that support the findings of this study are available from the corresponding author upon reasonable request.
