# Supplementary material for: Machine Learning‐Enhanced Optimization for High‐Throughput Precision in Cellular Droplet Bioprinting
Source: Adv Sci (Weinh). 2025 Apr 27;12(20):2412831. doi: 10.1002/advs.202412831 (PMC12120697; doi:10.1002/advs.202412831)
Supplement: Supplementary file 1 — Supporting Information [file ADVS-12-2412831-s006.docx]

**Supporting Information**

Machine Learning-Enhanced Optimization for High-Throughput Precision in Cellular Droplet Bioprinting

*Jaemyung Shin^1^, Ryan Kang^2^, Kinam Hyun^3^, Zhangkang Li^1^, Hitendra Kumar^4^, Kangsoo Kim^2^, Simon S. Park^3^, and Keekyoung Kim^1,3 *^*

^1^Department of Biomedical Engineering, Schulich School of Engineering, University of Calgary, Calgary, Alberta T2N 1N4, Canada

^2^Department of Electrical and Software Engineering, Schulich School of Engineering, University of Calgary, Calgary, Alberta T2N 1N4, Canada

^3^Department of Mechanical and Manufacturing Engineering, Schulich School of Engineering, University of Calgary, Calgary, Alberta T2N 1N4, Canada

^4^Department of Biosciences and Biomedical Engineering, Indian Institute of Technology Indore, Indore, Madhya Pradesh 453552, India

^*^**Corresponding author**: Dr. Keekyoung Kim, email: [keekyoung.kim@ucalgary.ca](mailto:keekyoung.kim@ucalgary.ca)


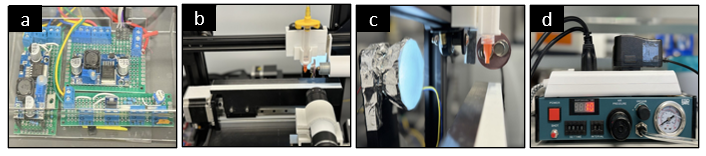


**Supplementary Fig. S1:** Components of the high-throughput 3D bioprinter and imaging system. **a.** A custom-designed printed circuit board for system control and bioprinting. **b** Motorized stage assembly for precise positioning and high-throughput image collection. **c** Optimized LED lighting configuration for consistent and uniform illumination while printing. **d** Liquid dispenser for accurate and controlled sample deposition.


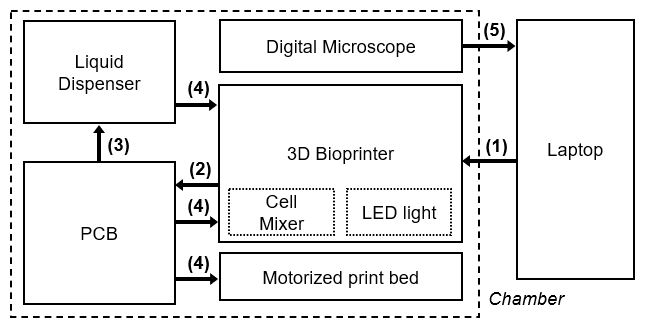


**Supplementary Fig. S2:** A comprehensive signal diagram illustrating the workflow of a whole bioprinting platform. The setup includes a laptop connected to a 3D bioprinter and a modified printed circuit board (PCB) integrated with a liquid dispenser. This interconnected system facilitates precise droplet dispensing and subsequent image collection, highlighting the seamless coordination between hardware components and software control for efficient bioprinting operations.


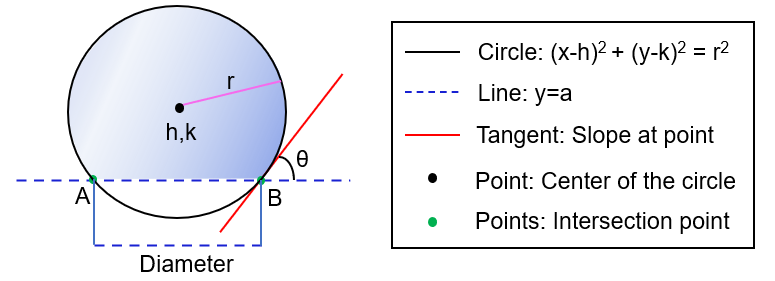


**Supplementary Fig. S3:** Schematic illustration to aid in understanding the method for automated droplet size calculation.

**
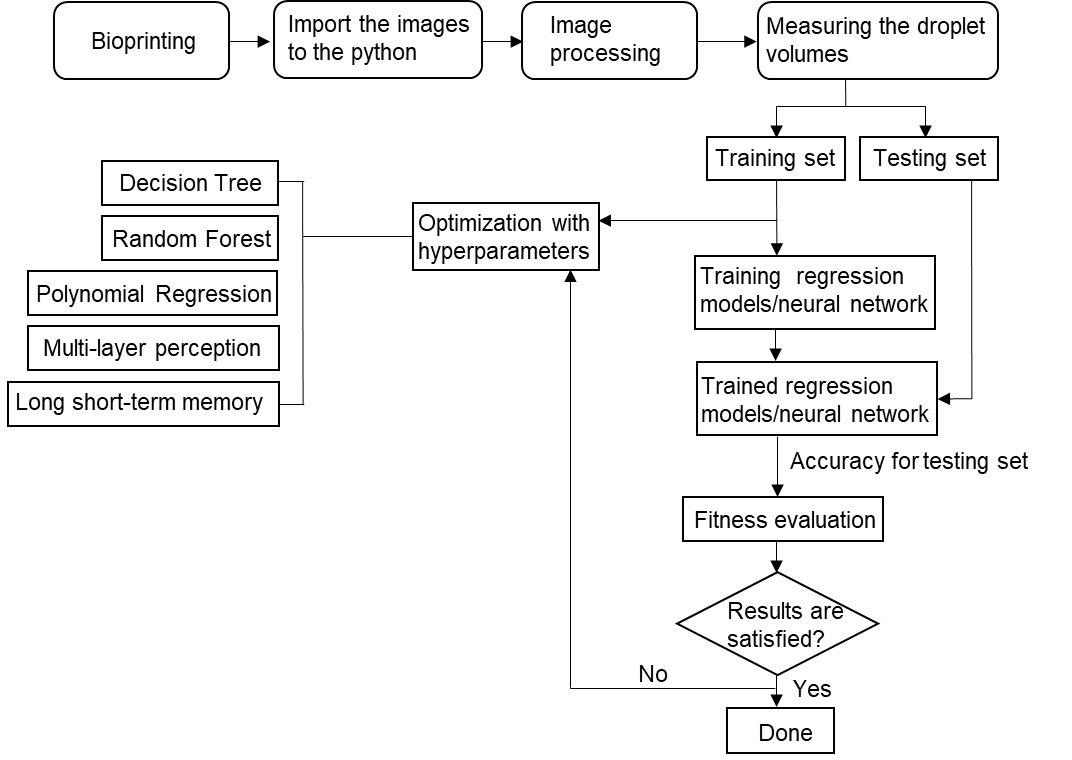
**

**Supplementary Fig. S4:** Integrated framework for bioprinting parameter optimization using traditional machine learning and deep learning algorithms.

**Supplementary Table S1.** Comparison between traditional machine learning and deep learning.

|  | **Traditional machine learning** | **Deep learning** |
| --- | --- | --- |
| **Data size** | Efficient with modest-sized datasets and suited for tasks of moderate complexity. | Excels on large and intricate datasets, suitable for high-dimensional and complex data. |
| **Complexity of model** | General and shallow model in a concise and formal tone. | Utilization of a multilayer neural network structure and complex model. |
| **Hardware and optimization** | Not specifically dependent on hardware, applicable to various architectures | Specific hardware (GPU, TPU) requirements and optimization techniques are necessary for efficient training. |
| **Batch processing** | Processing data in small batches | It is generally more suitable for processing in bulk batches to efficiently handle large-scale datasets. |
| **Hyperparameter adjustment** | Hyperparameters are typically manually tuned based on domain knowledge and experimentation. | Adjusting more hyperparameters requires utilizing automated methods for hyperparameter tuning. |

**Supplementary Table S2.** Statistical analysis of bioprinting parameter weights on droplet volume using the Tukey method with a 95% confidence interval

| **Parameter** | **N** | **Mean** | **Grouping** |
| --- | --- | --- | --- |
| Dispensing time | 5 | 0.387772 | A |
| Nozzle gauge | 5 | 0.257286 | B |
| Printing pressure | 5 | 0.196495 | C |
| Cell concentration | 5 | 0.114062 | D |
| Viscosity | 5 | 0.044384 | E |

*Means that do not share a letter are significantly different.*

**Supplementary Table S3.** Types of hyperparameters utilized in optimization before algorithm training: their functions and roles

| **Hyperparameter** | **Function** |
| --- | --- |
| n_estimators | number of trees |
| criterion | impurity indicators (gini, entropy, log_loss) |
| max_depth | maximum depth of tree |
| mim_samples_split | minimum number of samples required to split an internal node |
| min_samples_leaf | minimum number of samples a leaf node must have |

**Supplementary Movie S1**. High-throughput cellular microarray 3D bioprinting

**Supplementary Movie S2**. Bioprinting workflow on the integrated platform

**Supplementary Movie S3**. Bioprinting of droplets with a minimum volume of 0.1 µL

**Supplementary Movie S4**. Cell Stirring system in syringe to prevent cell sedimentation

**Supplementary Movie S5.** Crosslinking with 405 nm blue light post-printing

**Supplementary Movie S6.** Day 3 of the time-lapse movie of GFP-tagged 3T3 fibroblast cells encapsulated in 5% Gelma and 2% Alginate bioink droplets (~500 µm thickness) across different focal planes

**Supplementary Movie S7**. Bioprinting of 60 droplets on a single glass slide
